# Impaired muscle mitochondrial energetics is associated with uremic metabolite accumulation in chronic kidney disease

**DOI:** 10.1172/jci.insight.139826

**Published:** 2020-12-08

**Authors:** Trace Thome, Ravi A. Kumar, Sarah K. Burke, Ram B. Khattri, Zachary R. Salyers, Rachel C. Kelley, Madeline D. Coleman, Demetra D. Christou, Russell T. Hepple, Salvatore T. Scali, Leonardo F. Ferreira, Terence E. Ryan

**Affiliations:** 1 Department of Applied Physiology and Kinesiology, College of Health & Human Performance,; 2 Department of Physical Therapy, College of Public Health and Health Professions,; 3 Center for Exercise Science, College of Health & Human Performance, and; 4 Division of Vascular Surgery and Endovascular Therapy, Department of Surgery, College of Medicine, University of Florida, Gainesville, Florida, USA.; 5 Malcom Randall Veterans Affairs Medical Center, Gainesville, Florida, USA.

**Keywords:** Muscle Biology, Nephrology, Bioenergetics, Mitochondria, Skeletal muscle

## Abstract

Chronic kidney disease (CKD) causes progressive skeletal myopathy involving atrophy, weakness, and fatigue. Mitochondria have been thought to contribute to skeletal myopathy; however, the molecular mechanisms underlying muscle metabolism changes in CKD are unknown. We employed a comprehensive mitochondrial phenotyping platform to elucidate the mechanisms of skeletal muscle mitochondrial impairment in mice with adenine-induced CKD. CKD mice displayed significant reductions in mitochondrial oxidative phosphorylation (OXPHOS), which was strongly correlated with glomerular filtration rate, suggesting a link between kidney function and muscle mitochondrial health. Biochemical assays uncovered that OXPHOS dysfunction was driven by reduced activity of matrix dehydrogenases. Untargeted metabolomics analyses in skeletal muscle revealed a distinct metabolite profile in CKD muscle including accumulation of uremic toxins that strongly associated with the degree of mitochondrial impairment. Additional muscle phenotyping found CKD mice experienced muscle atrophy and increased muscle protein degradation, but only male CKD mice had lower maximal contractile force. CKD mice had morphological changes indicative of destabilization in the neuromuscular junction. This study provides the first comprehensive evaluation of mitochondrial health in murine CKD muscle to our knowledge and uncovers several unknown uremic metabolites that strongly associate with the degree of mitochondrial impairment.

## Introduction

Chronic kidney disease (CKD) affects more than 25 million Americans (1). Unfortunately, CKD has no cure and available treatment options for end-stage renal disease are limited to kidney transplant or chronic hemodialysis. A hallmark of the CKD phenotype is the presentation of debilitating myopathic symptoms, including muscle weakness, fatigue, and muscle wasting, which inhibit functional independence and quality of life, contributing to increased morbidity and mortality (2). Loss of muscle mass in CKD has been attributed to imbalances in protein synthesis and protein degradation pathways (3), resulting in a cachectic phenotype. Consistent with this idea, previous studies have demonstrated activation of proteolytic processing, including ubiquitin-proteasome pathway, caspase-3 and other atrophy signaling pathways, as well as myostatin, in CKD (4–9). The activation of pathways contributing to muscle loss has been largely attributed to the multitude of CKD complications, including metabolic acidosis, inflammation, elevated glucocorticoid levels, and impaired IGF-1 signaling. Recently, evidence for crosstalk between the kidneys and skeletal muscle has garnered support (10, 11), suggesting systemic factors directly link kidney function to skeletal muscle health in CKD.

Several studies have implicated skeletal muscle mitochondria as a contributor to CKD-associated myopathy (12–18). Studies in animal models and human CKD muscle commonly observe decreased mitochondrial content (12, 14, 19), impaired mitochondrial respiration (12, 18, 20, 21), and elevated oxidative stress (22, 23). Because mitochondria are the primary site for ATP production and generation of the free energy for ATP hydrolysis (i.e., capacity to power cellular work), impairment in mitochondrial energy transduction is likely a significant factor determining physical function and exercise tolerance in CKD patients. Indeed, recent clinical studies have confirmed that muscle oxidative capacity, measured by magnetic resonance spectroscopy, is strongly associated with physical performance in CKD patients (21, 24). Despite these reports, a comprehensive understanding of how impaired kidney function alters skeletal muscle mitochondrial health is lacking. To address this significant knowledge gap, we applied a comprehensive mitochondrial phenotyping protocol to elucidate specific sites of mitochondrial impairments in mice with CKD and performed untargeted metabolomics in muscle to uncover global metabolic changes in CKD muscle. Additionally, deep muscle phenotyping, including assessments of muscle atrophy and protein degradation, as well as contractile function and neuromuscular junction morphology, were performed to fully characterize the myopathy.

## Results

### Adenine-induced CKD produces a clinically relevant phenotype.

**Figure 1:**
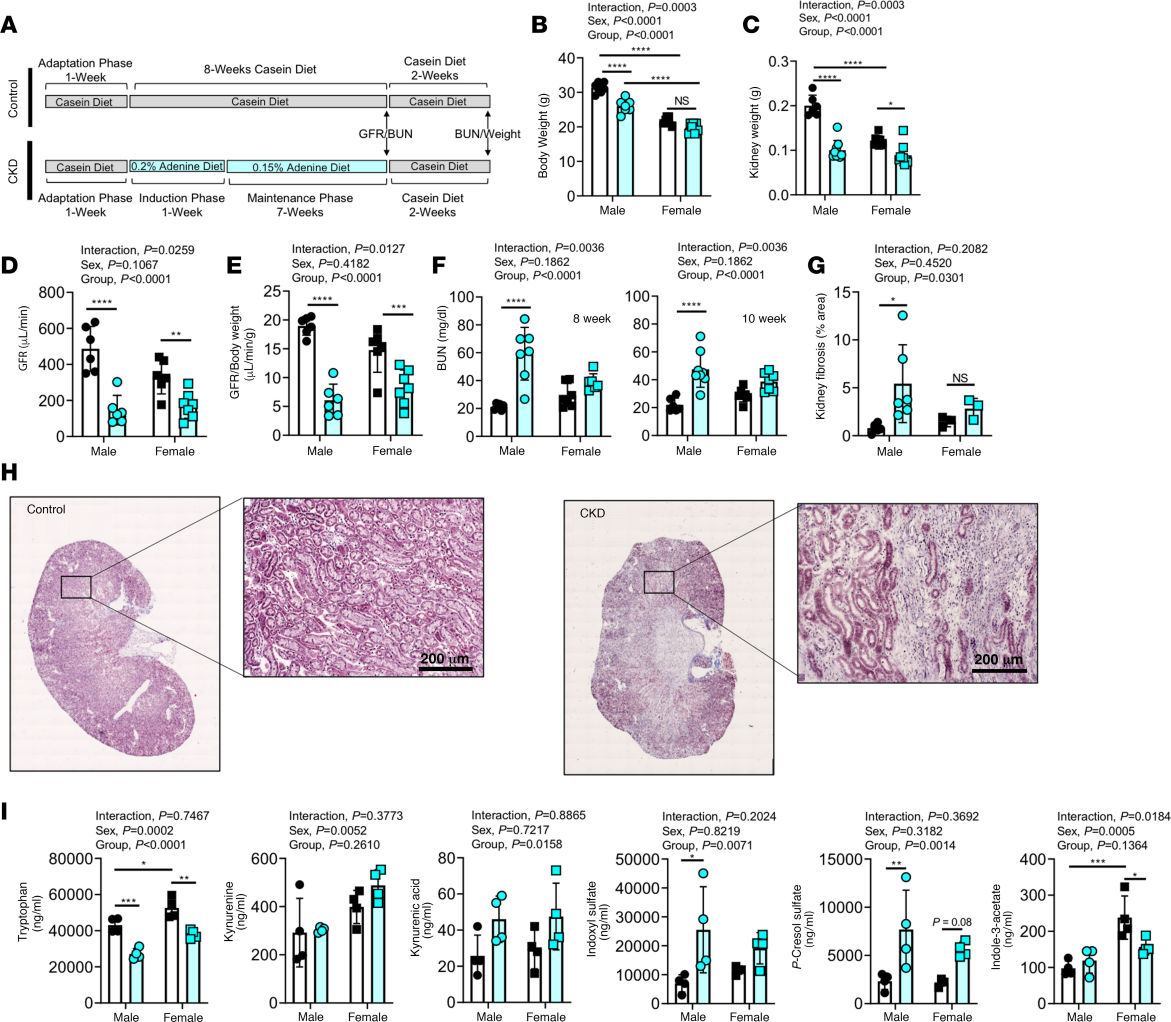
Adenine diet causes CKD with uremia. (**A**) Experimental design for diet manipulation and timing of relevant outcome measures. (**B**) Quantification of body weights in control and CKD mice (*n* = 7–8/group/sex). (**C**) Quantification of kidney weights (*n* = 6–9/group/sex). (**D**) Glomerular filtration rate (GFR) measured by FITC-inulin clearance (*n* = 6–8/group/sex). (**E**) GFR results normalized to body weight (*n* = 6–7/group/sex). (**F**) Blood urea nitrogen (BUN) measured after 8 weeks on adenine diet and 10 weeks following 2 week washout before sacrifice (*n* = 6–8/group/sex). (**G**) Quantification of kidney fibrotic area (*n* = 3–6/group/sex). (**H**) Representative histological images (Masson’s trichrome) of control and CKD kidneys. (**I**) Serum metabolomics analysis for selected uremic toxins measured at sacrifice (*n* = 4/group/sex). *P < 0.05, **P < 0.01, ***P < 0.001, ****P < 0.0001 using 2-way ANOVA and Tukey’s post hoc test when appropriate. Error bars show standard deviation. Scale bars: 200 μm.

Supplementing the diet with adenine is an established model for inducing CKD in rodents (25–29) that results in tubulointerstitial nephropathy caused by increased production and subsequent crystallization of 2,8-dihydroxyadenine in renal tubules that cause kidney damage. In this study, mice that received adenine-supplemented diet for 8 weeks were subsequently placed back on casein feed to allow removal of any potential side effects of adenine (Figure 1A). Adenine feeding resulted in lower body weights in male but not female mice (Figure 1B), despite significant reductions in glomerular filtration rate (GFR) (both absolute and normalized to body weight) in both sexes (Figure 1, D and E). Surprisingly, blood urea nitrogen (BUN) measured both at 8 weeks (end of adenine diet) and 10 weeks (sacrifice) was significantly elevated in male, but not female, CKD mice (Figure 1F). Importantly, placing mice back on casein diet for 2 weeks before sacrifice did not affect BUN (Figure 1F), confirming little recovery in kidney function. CKD mice displayed clinically relevant signs of pathology, including smaller kidney weight (Figure 1C) with cyst-like appearance (Figure 1H), as well as greater fibrotic tissue area and evidence of interstitial inflammation (Figure 1, G and H). A recognized phenotype of CKD patients is the development of uremia, characterized by increased levels of uremic metabolites (toxins) in the blood (30–32). Targeted serum metabolomics analyses confirmed elevations in commonly reported tryptophan-derived uremic toxins, including kynurenic acid, indoxyl sulfate, and *p*-cresol sulfate (Figure 1I). Taken together, these pathological characteristics support the relevance of an adenine model to study CKD in a preclinical setting.

### CKD skeletal muscle exhibits reduced oxidative capacity and OXPHOS conduction.

**Figure 2:**
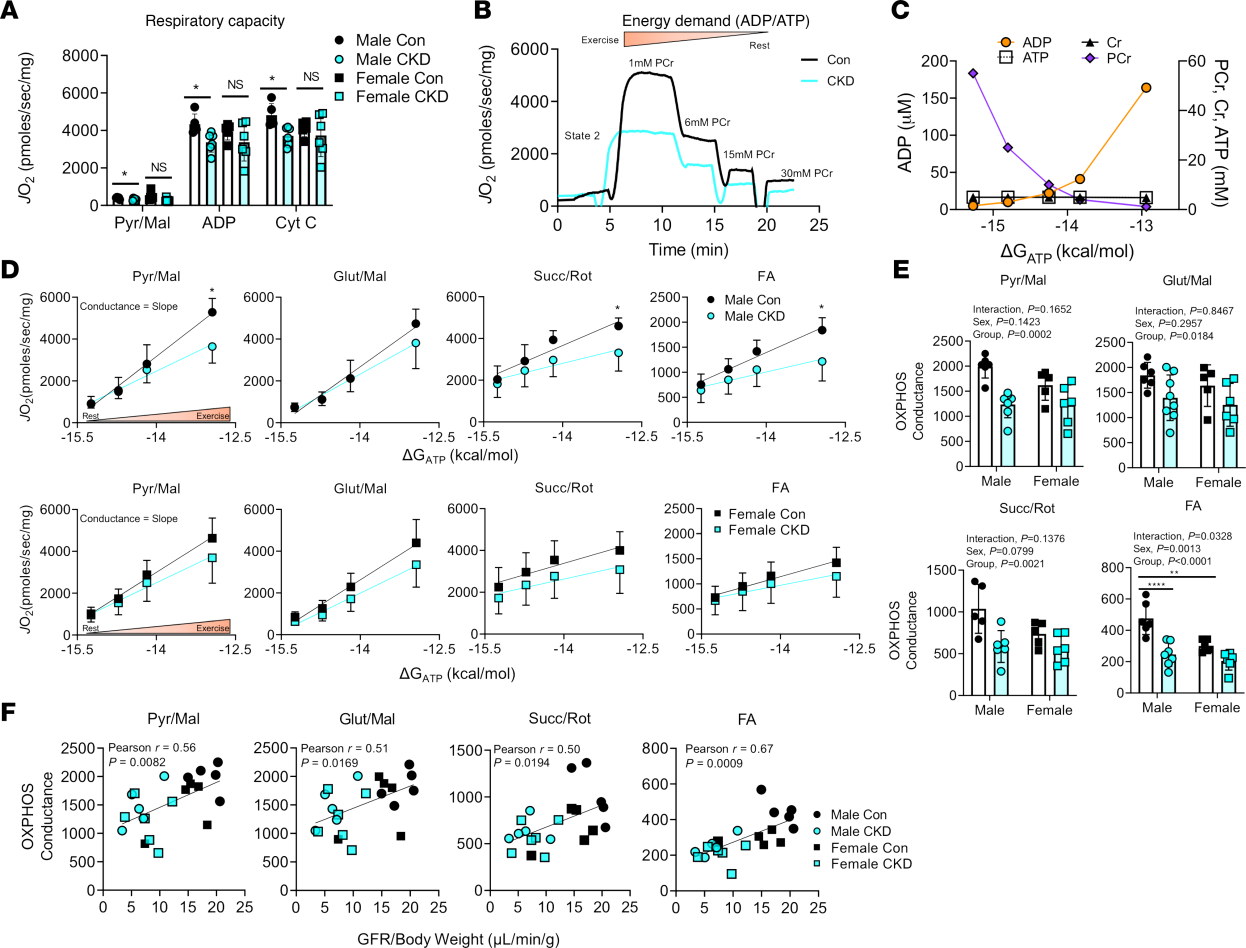
CKD impairs mitochondrial energy transduction and correlates with kidney function. Skeletal muscle mitochondria were isolated from control and CKD mice. (**A**) Maximal ADP-stimulated respiration supported by pyruvate and malate (Pyr/Mal) was significantly decreased in male CKD mice (*n* = 5–6/group/sex). (**B**) Next, a physiological assessment of mitochondrial OXPHOS was employed using a creatine kinase clamp (**C**) to establish physiologically relevant levels of extramitochondrial energy demand (i.e., mimic a stress test). (**D**) Male and female mitochondrial oxygen consumption (*J*O_2_) plotted against energy demand (ΔG_ATP_) for the following substrate conditions: pyruvate + malate, glutamate + malate, succinate + rotenone, and fatty acid (FA, octanoyl-carnitine + malate) (*n* = 5–7/group/sex). (**E**) Quantification of OXPHOS conductance for each substrate condition (slope of *J*O_2_ vs. ΔG_ATP_) (*n* = 5–7/group/sex). (**E**) Pearson correlation analysis of OXPHOS conductance and GFR demonstrate a strong association between kidney function and mitochondrial OXPHOS in skeletal muscle. Data were analyzed by multiple 2-tailed Student’s *t* test (**D**) or 2-way ANOVA with Tukey’s post hoc testing when an interaction was detected (**A** and **E**). Error bars show standard deviation. *P < 0.05, **P < 0.01, and ****P < 0.0001. NS, not significant.

To assess the impact of CKD on skeletal muscle mitochondrial energetics, oxidative capacity and a physiological assessment of the oxidative phosphorylation (OXPHOS) system were measured using multiple substrate combinations. Using classic oxidative capacity assessments, isolated mitochondria were energized with pyruvate/malate followed by the addition of a bolus of ADP (4 mM). Saturating concentrations of ADP override the mitochondrial internal mechanism of responding to energy demand and trigger state 3 levels of respiration. Male CKD mice had approximately 22% decrease in maximal respiration compared with controls (*P* < 0.05), while female CKD mice displayed a more modest approximately 14% decrease in maximal respiration (Figure 2A). To provide a more comprehensive and physiologically relevant assessment of mitochondrial function, we employed an approach in which the extramitochondrial energy demand (ATP/ADP) is controlled using a creatine kinase clamp system (33–36). This system allows for assessment of mitochondrial oxygen consumption (*J*O_2_) at varying levels of energy demand (ΔG_ATP_) that correspond to physiologically relevant levels of cellular ATPase activity (Figure 2, B and C). Plotting the respiratory flux as a function of ΔG_ATP_ allows for calculation of conductance (slope of the linear relationship) of the mitochondrial energy transduction system (high conductance = good function). Using 4 fuel sources, carbohydrate (pyruvate/malate, glutamate/malate, succinate/rotenone) and fatty acid (FA) (octanoyl-carnitine/malate) substrates, OXPHOS conductance was reduced in male and female CKD mice 24%–42% (*P* < 0.05 for all substrates) in muscle mitochondria (Figure 2, D and E). Pearson correlation analyses demonstrated a strong direct relationship between muscle mitochondrial OXPHOS conductance and kidney function (GFR) across all substrate conditions (Figure 2F). This observation supports a uremic myopathy hypothesis whereby uremic metabolites not adequately filtered by the kidneys disrupt mitochondrial metabolism (17, 34).

### Mitochondrial H_2_O_2_ production and electron leak are minimally affected by CKD.

**Figure 3:**
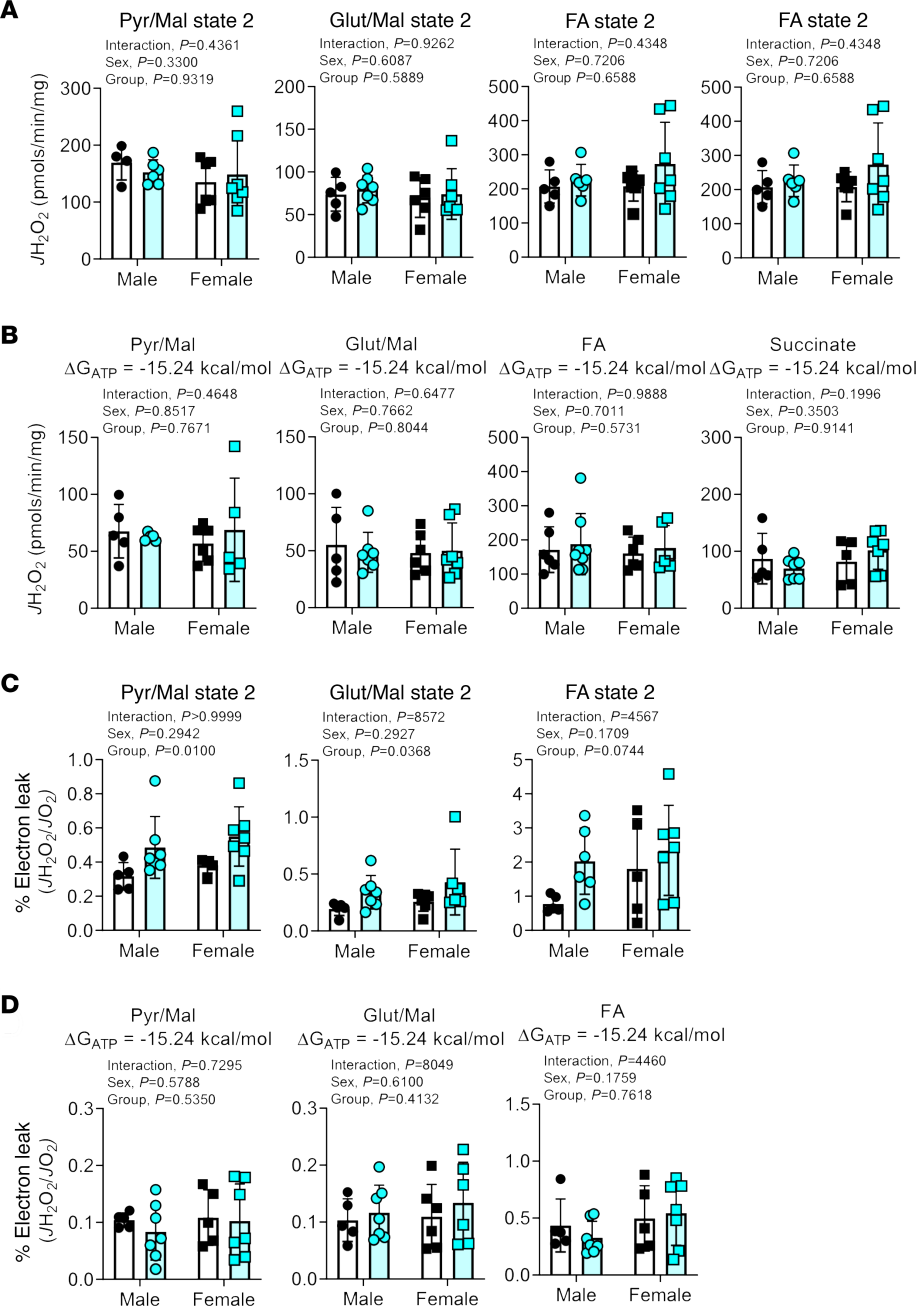
CKD does not alter skeletal muscle mitochondrial H_2_O_2_ production or electron leak under physiological conditions. (**A**) Mitochondrial H_2_O_2_ production (*J*H_2_O_2_) under state 2 (no ADP = no energy demand) for the following substrates: pyruvate + malate, glutamate + malate, FA + malate, and succinate. (**B**) Mitochondrial H_2_O_2_ production levels under conditions mimicking resting energy demand (ΔG_ATP_ = –15.24 kcal/mol) for each substrate condition. (**C**) Calculated percentage electron leak (*J*H_2_O_2_/*J*O_2_) for state 2 respiration for the following substrates (pyruvate + malate, glutamate + malate, FA + malate). Succinate was not used due to the presence of rotenone during the OXPHOS conductance experiments. (**D**) Calculated percentage electron leak (*J*H_2_O_2_/*J*O_2_) under resting level energy demand (ΔG_ATP_ = –15.24 kcal/mol) for each substrate condition. All data were analyzed using 2-way ANOVA followed by Tukey’s post hoc test when an interaction was established. Error bars show standard deviation.

The lower OXPHOS conductance in CKD muscle mitochondrial indicates there is more “resistance” in mitochondrial energy transfer. Increased resistance of this bioenergetic system could manifest as increased levels of ROS. Thus, mitochondrial H_2_O_2_ production was assessed using Amplex UltraRed in combination with the creatine kinase clamp system to determine H_2_O_2_ production at state 2 (no energy demand) and resting levels of energy demand (ΔG_ATP_ = –15.24 kcal/mol). Interestingly, mitochondrial H_2_O_2_ production under state 2 and resting conditions were not different between control and CKD groups for all substrate conditions (Figure 3, A and B). For pyruvate/malate, glutamate/malate, and octanoyl-carnitine/malate conditions, an estimation of electron leak can be calculated by dividing the *J*H_2_O_2_ by the corresponding *J*O_2_ under identical assay conditions. The calculated electron leak was greater in CKD mitochondria for pyruvate/malate and glutamate/malate (*P* *<* 0.05) and was trending with octanoyl-carnitine/malate substrates under state 2 conditions (*P* = 0.0744) (Figure 3C). Notably, these mild elevations in electron leak disappeared when the mitochondria were placed under resting levels of energy demand (Figure 3D), suggesting that elevated mitochondrial ROS production is not a characteristic of CKD muscle, at least during the relatively short durations (~10 weeks) employed herein.

### Skeletal muscle mitochondrial content is normal in adenine-fed mice.

**Figure 4:**
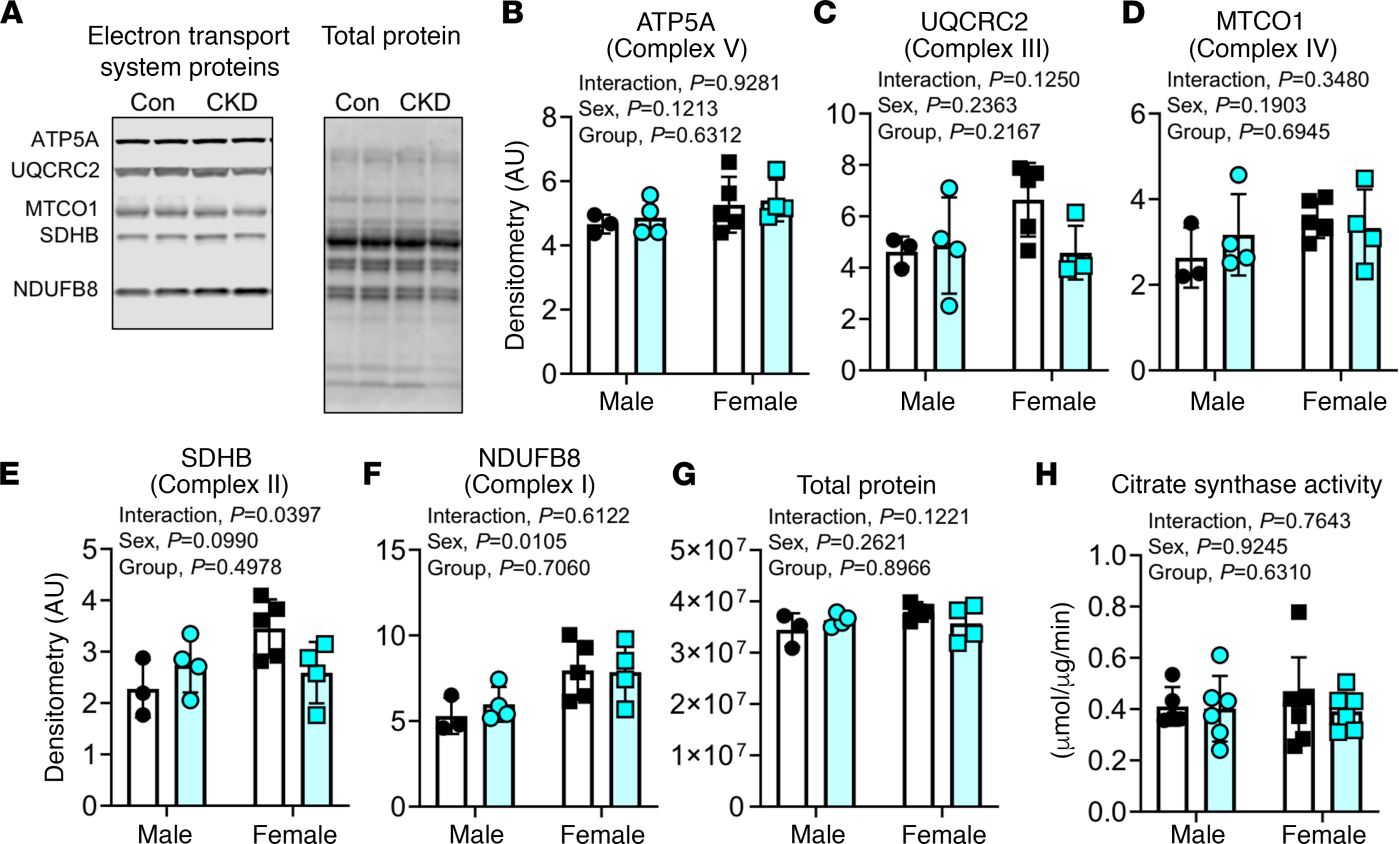
Adenine-induced CKD did not change mitochondrial content in skeletal muscle. (**A**) Representative Western blotting for select protein subunits of the electron transport system (ETS) in individual mitochondrial enzyme complexes along with corresponding total protein imaging. (**B**–**F**) Quantification of protein abundance from Western blotting using standard densitometry and normalization to each lane’s protein value (*n* = 3–4/group/sex). (**G**) Quantification of total protein in all samples confirms even loading and transfer (*n* = 3–4/group/sex). (**H**) Citrate synthase activity measured in whole gastrocnemius muscle lysates further confirms similar mitochondrial content in skeletal muscle of CKD mice (*n* = 5–7/group/sex). All data were analyzed using 2-way ANOVA with Tukey’s post hoc test when an interaction was detected. Error bars show standard deviation.

Several studies examining the effects of CKD on mitochondria have demonstrated a reduction in mitochondrial content in skeletal muscle of rodents and humans with CKD (33–36). Assessments of citrate synthase enzyme activity and protein abundance of electron transport system (ETS) protein subunits were performed in red gastrocnemius muscle lysates. There was no difference in either the protein content of selected ETS protein subunits assessed by Western blotting (Figure 4, A–G) or citrate synthase activity (Figure 4H) between control and CKD mice.

### Impaired matrix dehydrogenase activity in CKD skeletal muscle.

**Figure 5:**
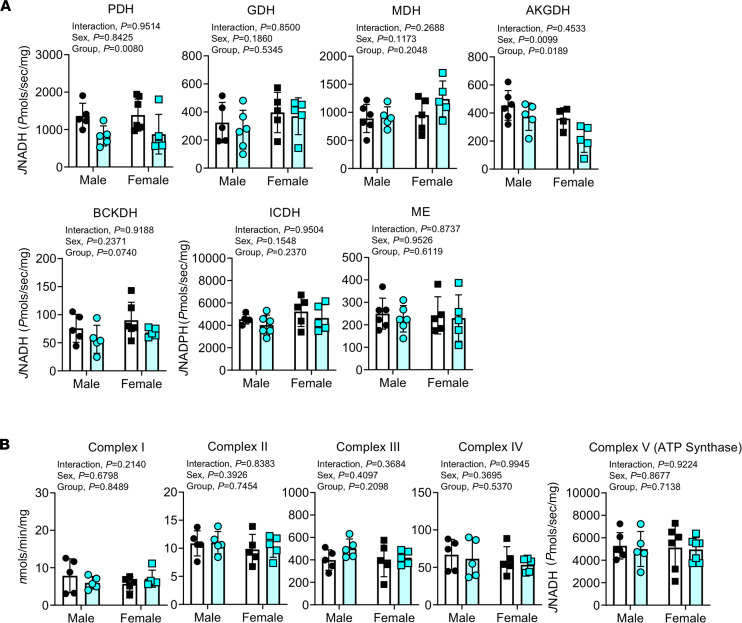
CKD reduces activity of select matrix dehydrogenases but does not impair ETS enzyme activity. Skeletal muscle mitochondria were isolated from control and CKD mice and subsequently permeabilized using alamethicin (0.03 mg/mL) or were lysed in cell lytic and underwent freeze thaw cycles for complex enzymatic assays (complexes I–V). (**A**) Matrix dehydrogenase activity was measured for the following enzymes: pyruvate dehydrogenase (PDH), glutamate dehydrogenase (GDH), malate dehydrogenase (MDH), alpha ketoglutarate dehydrogenase (AKGDH), branched chain keto acid dehydrogenase (BCKDH), isocitrate dehydrogenase (ICDH), and malic enzyme (*n* = 5–7/group/sex). (**B**) Enzyme activity of the complexes of the ETS was assessed using standard biochemical assays (*n* = 6–7/group/sex). All data were analyzed using a 2-way ANOVA. Error bars show standard deviation.

To elucidate the specific sites responsible for the decreased OXPHOS in CKD muscle, a series of biochemical enzyme assays were performed to assess the function of numerous matrix dehydrogenases, the enzyme complexes of the ETS, and the ATP synthase. These 3 groups of enzymes are considered the major control nodes of mitochondrial energy transfer (fuel **→** redox potential **→** proton motive force **→** ΔG_ATP_). CKD mice exhibited significant decreases (approximately 35%) in the activity of pyruvate dehydrogenase (PDH) and (approximately 27%) in alpha ketoglutarate dehydrogenase (AKGDH) compared with control mice (Figure 5A). Moreover, BCKDH displayed a reduction in activity that approached, but did not reach, statistical significance (*P* = 0.0740) (Figure 5A). Next, traditional biochemical assays were performed to assess the enzyme activities of the ETS. Results from these experiments indicated no difference in any of the ETS complex enzyme activity (complexes I–IV) (Figure 5B) or the ATP synthase (complex V) between CKD and control mice. These results indicate that mitochondrial impairments in the skeletal muscle of CKD mice are derived primarily from impaired dehydrogenase function.

### CKD mice display distinct metabolite profiles in skeletal muscle.

**Figure 6:**
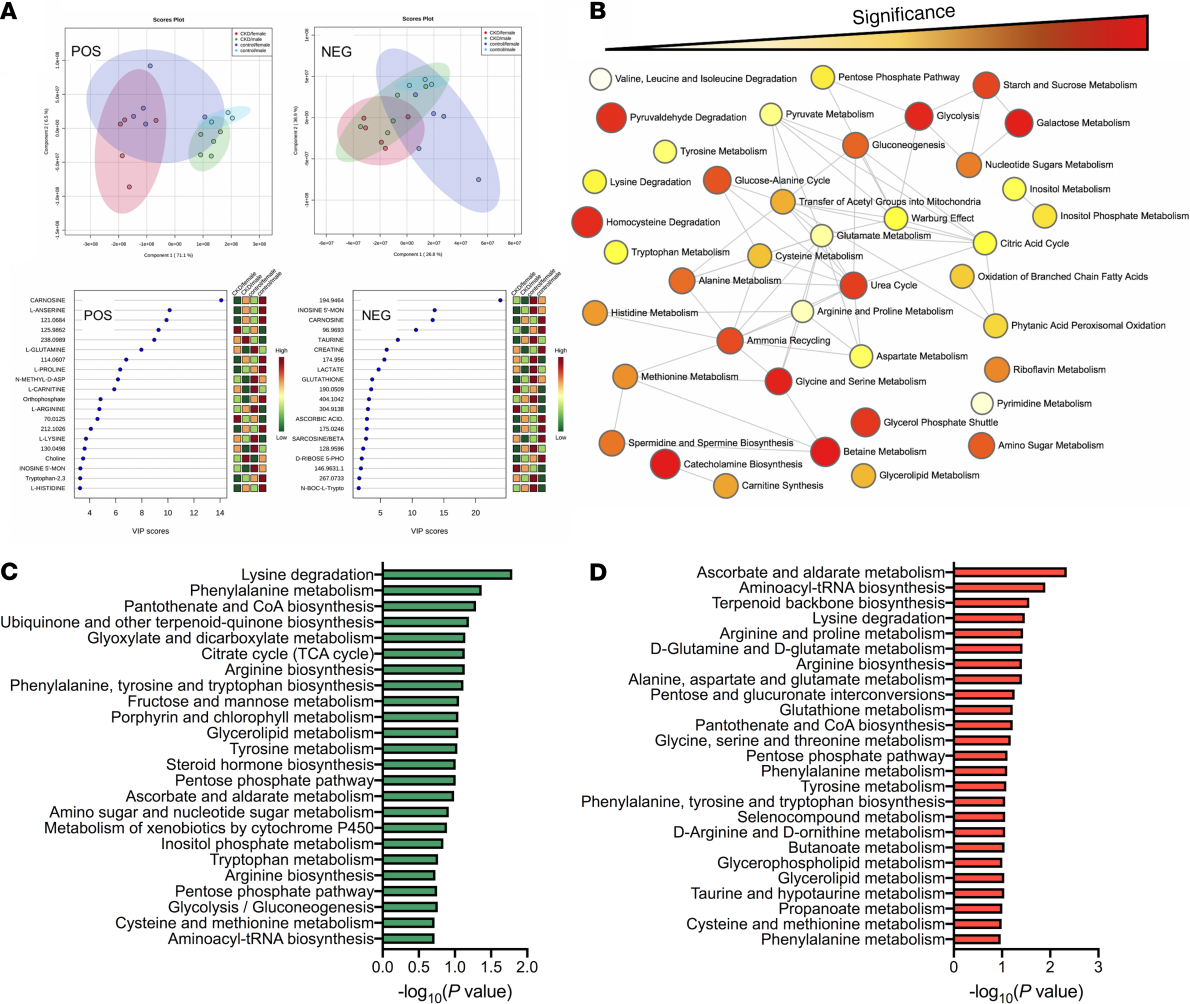
CKD results in changes to the skeletal muscle metabolome. Untargeted metabolomics analysis was performed in red gastrocnemius muscle of control and CKD mice (*n* = 3 control males, *n* = 5 CKD males, *n* = 5 females/group). (**A**) Principal component analysis and partial least squares discriminant analysis with the top 20 variable importance in projection–scored (VIP-scored) metabolites for positive and negative ion mode results. (**B**) Enrichment network analysis was performed using MetaboAnalyst 4.0 using combined sexes (control vs. CKD). Color represents level of significance; size of circle represents fold enrichment (hits/expected). (**C** and **D**) KEGG pathway analysis of differentially expressed metabolites in (**C**) male and (**D**) female CKD mice.

To understand metabolic changes in the skeletal muscle of mice with CKD, global (untargeted) metabolomics analyses were performed in gastrocnemius muscle. Following metabolites’ extraction, samples were processed in both positive and negative ion modes and analyzed separately. A total of 1832 features were detected in positive ion mode, whereas 1320 features were detected in negative ion mode. Features were identified using an internal retention time library. As with many global metabolomics analyses, a number of features detected were not in our internal library and therefore cannot be identified. Principal component analysis across the 4 groups (male control, male CKD, female control, female CKD) indicated distinct grouping based on treatment (control vs. CKD) as well as sex (male vs. female) in both ion modes (Figure 6A). Partial least squares discriminant analysis and identification of the metabolites with the top 20 VIP scores are shown in Figure 6A. Because the metabolomics analyses were not powered to test for sex differences, we performed 1-way ANOVA comparing control with CKD within each sex using a *P* < 0.05 to identify statistically different metabolite features within each sex. A total of 204 and 111 metabolites in positive and negative ion mode, respectively, were statistically different in male mice. A total of 131 and 138 metabolites in positive and negative mode, respectively, were statistically different in female mice. A complete list of untargeted metabolomics features can be found in Supplemental Table 2; supplemental material available online with this article; https://doi.org/10.1172/jci.insight.139826DS1 Enrichment network analysis of differential metabolites in CKD muscle was performed using MetaboAnalyst 4.0 (Figure 6B). Notable network features enriched in CKD muscle involved carbohydrate metabolism, the urea cycle, and amino acid metabolism. Kyoto Encyclopedia of Genes and Genomes (KEGG) pathway analysis of differentially abundant metabolites in male and female CKD mice is shown in Figure 6, C and D, respectively.

**Figure 7:**
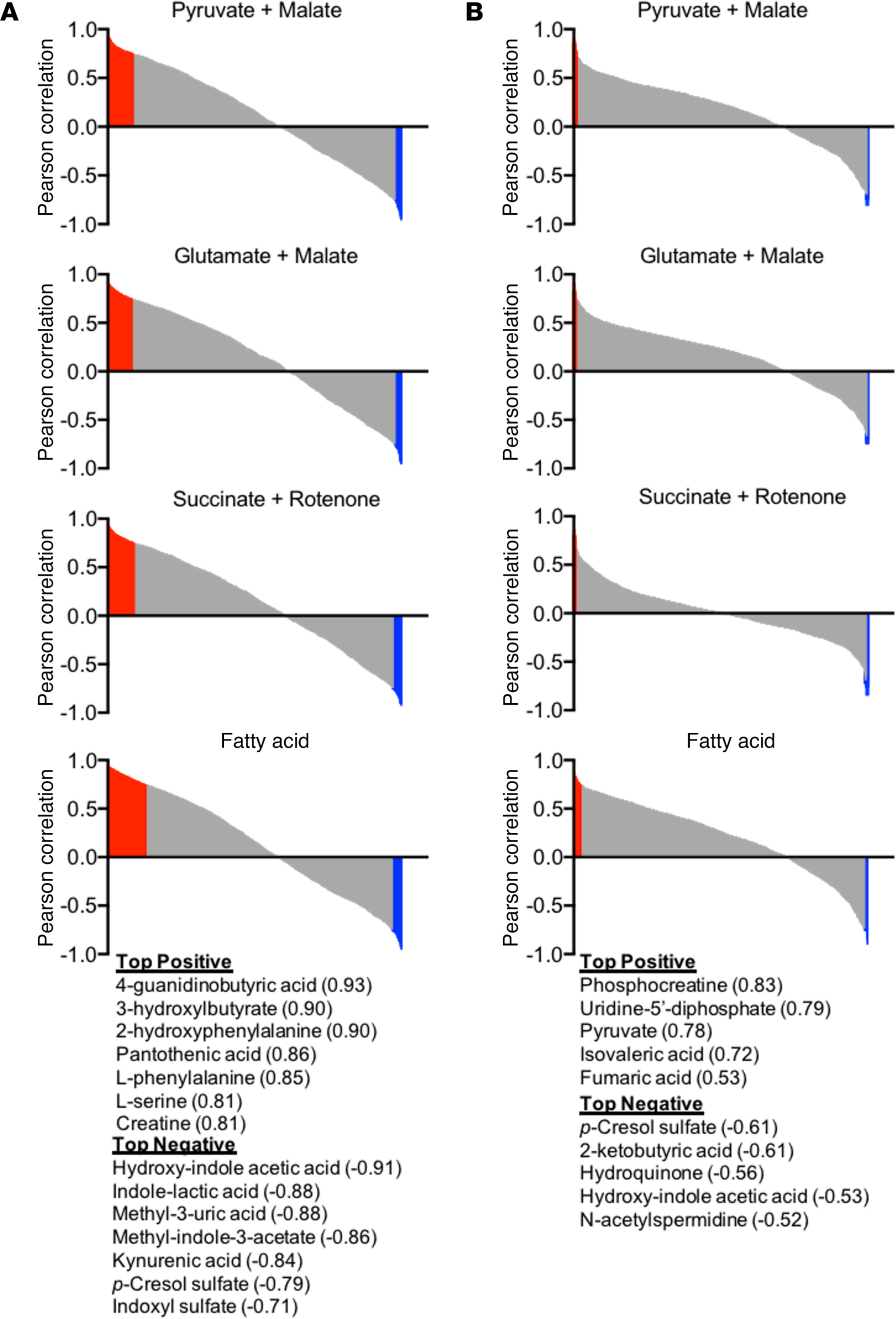
Accumulation of uremic metabolites in muscle associates with the degree of mitochondrial impairment. (**A**) Pearson correlations between metabolite concentration (peak area) and OXPHOS conductance for male mice (*n* = 3 control, *n* = 5 CKD). (**B**) Pearson correlations between metabolite concentration (peak area) and OXPHOS conductance for female mice (*n* = 5 control, *n* = 5 CKD). Metabolites with Pearson correlations with a *P* < 0.1 are shown in red (positive) or blue (negative). Top positive and negative correlating metabolites are listed by name and their corresponding Pearson correlation coefficient. Metabolite identities were determined using MetaboAnalyst 4.0.

Previous work has reported that some uremic metabolites can impair mitochondrial function (34, 37–40). Based on these observations, Pearson correlation analyses were performed to identify metabolite features that were highly correlated with OXPHOS conductance (negatively and positively). These analyses identified numerous metabolite features that were either negatively or positively correlated with OXPHOS conductance (Figure 7). Metabolite features in red or blue color exhibited a *P* < 0.1 for the Pearson correlation analysis. Top metabolite identities are also listed with their corresponding Pearson correlation coefficient. Interestingly, the number of metabolite features that associated with the OXPHOS kinetics was substantially greater in male mice (Figure 7A) compared with female mice (Figure 7B). However, many metabolites features that were highly abundant and associated with mitochondrial impairment in CKD were not directly identified using our internal metabolite library. Thus, future work is warranted to elucidate the chemical identity of these metabolites in order to facilitate testing the causative potential in uremic myopathy.

### Adenine-induced CKD causes increased muscle protein degradation and muscle atrophy.

**Figure 8:**
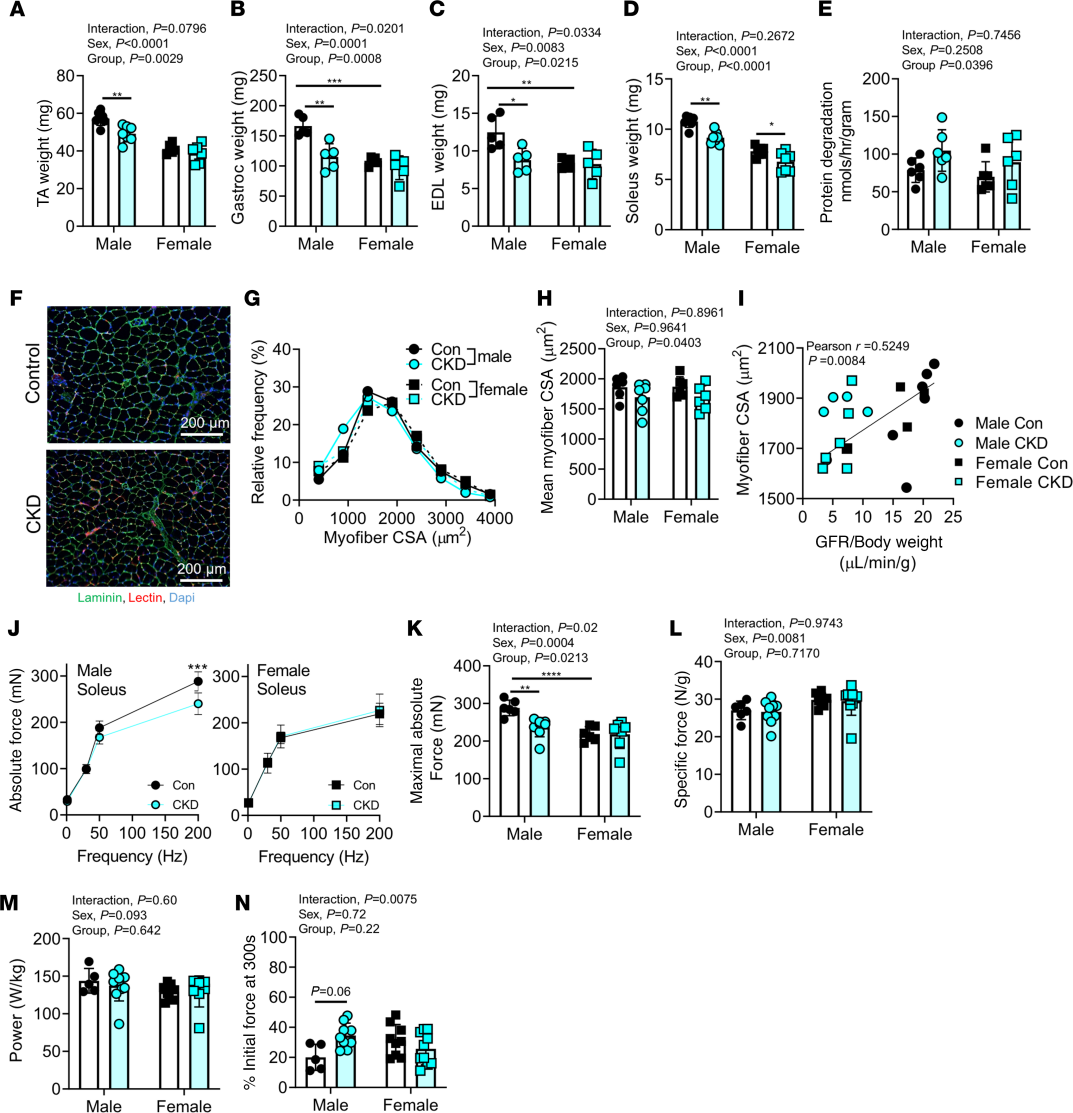
Adenine-induced CKD causes atrophy and increased protein degradation in muscle but does not alter muscle-specific force. (**A**–**D**) Quantified muscle weights for (**A**) TA, (**B**) gastrocnemius, (**C**) EDL, and (**D**) soleus muscles of control and CKD mice (*n* = 5–7/group/sex). (**E**) Protein degradation rates of soleus muscles in control and CKD mice (*n* = 6/group/sex). (**F**) Representative immunofluorescence images of the soleus muscle sections stained with laminin (myofibers), GS-lectin (capillaries), and DAPI (nuclei). (**G**) Histogram of soleus myofibers CSAs indicates a leftward shift in CKD mice (indicative of atrophy) (*n* = 6–7/group/sex). (**H**) Mean soleus myofibers CSA (*n* = 6–7/group/sex). (**I**) Pearson correlation between mean myofibers CSA and GFR across groups. (**J**) An abbreviated force frequency curve was used to assess soleus muscle force production ex vivo. Quantified peak absolute force (**K**) and specific force (**L**) (*n* = 5–10/group/sex). (**M**) Muscle power was quantified using a shortening contraction (*n* = 5–10/group/sex). (**N**) Muscle fatigue was assessed and quantified using repeated tetanic contractions (1/s) for 5 minutes (*n* = 5–10/sex/group). *P < 0.05, **P < 0.01, ***P < 0.001, and ****P < 0.0001 using 2-way ANOVA and Tukey’s post hoc when appropriate. Error bars show standard deviation. Scale bars: 200 μm.

In addition to the comprehensive mitochondrial phenotyping above, the functional impact on skeletal muscle was assessed. Muscle atrophy is commonly reported in both rodent models (8, 9, 41) and human patients (42–44) with CKD. Interestingly, significant group effects were observed in the muscle wet weights of the tibialis anterior (TA), gastrocnemius, soleus, and extensor digitorum longus (EDL) muscles (lower weights in CKD animals) (Figure 8, A–D). Interactions were also detected in gastrocnemius and EDL muscles. Therefore, post hoc testing was performed, which allowed us to detect the specific interaction where female CKD mice did not display any significant reductions in muscle mass relative to control females in both muscles. Moreover, rates of protein degradation in soleus muscles of control and CKD mice were measured ex vivo via quantification of tyrosine release. In this regard, CKD mice had an approximately 25% increase in the rate of protein degradation compared with control mice (*P* = 0.0396, Figure 8E). Further to this, analysis of myofiber cross-sectional area (CSA) in solei muscle supported significantly smaller myofiber CSA in CKD mice (group effect *P* = 0.0403) (Figure 8, F–H). Similar to mitochondrial OXPHOS, a significant correlation (Pearson *r* = 0.5249, *P* = 0.00084) was found between the mean myofiber CSA and GFR across groups (Figure 8I). Correlations between the myofiber CSA remained statistically significant (*P* < 0.05) when performed within each sex.

While muscle atrophy is commonly reported in rodent studies of CKD, muscle contractile function is rarely assessed. To examine the functional impact of the uremic condition, the soleus muscle was excised and carefully sutured to a force transducer, and ex vivo assessments of force production, power, and fatigue were performed. A significant sex × group interaction was detected in peak absolute force (*F*_1,23_ = 5.756, *P* = 0.0249) (Figure 8, J and K). Post hoc testing indicated a significant decrease in peak absolute force in male CKD mice (288 ± 21 vs. 237 ± 26 mN, *P* = 0.0098) but not female CKD mice (219 ± 20 vs. 218 ± 38 mN, *P* = 0.9999). Interestingly, when normalized to muscle weight, specific force was not different between control and CKD mice in either sex (Figure 8L), demonstrating that in this study the decrease in absolute force in male CKD mice is driven by atrophy and not impairments of the contractile apparatus. Moreover, additional functional assessments demonstrated that CKD did not affect peak power or the rate of fatigue development (Figure 8, M and N).

### CKD mice have altered neuromuscular junction morphology.

**Figure 9:**
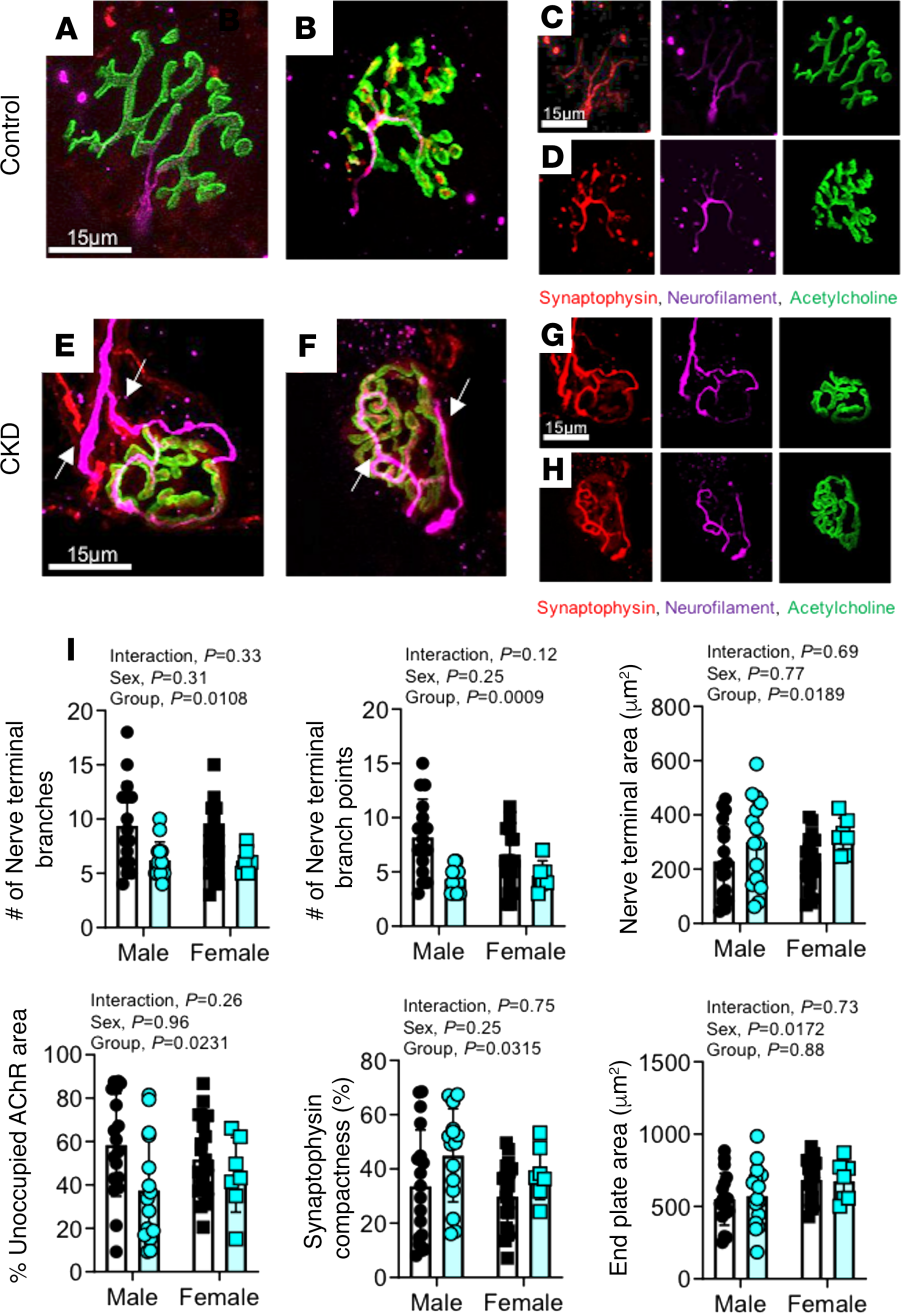
Evidence of NMJ remodeling in CKD mice. Representative immunofluorescence images of the NMJ morphology in TA muscle from control (**A**–**D**) and CKD mice (**E**–**H**). Images in panels **C**, **D**, **G**, and **H** are individual channels whereas **A**, **B**, **E**, and **F** are merged images. White arrows (**E** and **F**) demonstrate polyinnervation indicative of NMJ remodeling. NMJ labeling was performed using antibodies against the acetylcholine receptor (AChR), synaptophysin (terminal motor neuron), and neurofilament 200 (motor neuron axon). (**I**) Morphological analysis was performed by a blinded investigator for the following: number of nerve terminal branches and branch points, nerve terminal area, percentage area of AChR unoccupied, compactness of synaptophysin, and motor end plate area (*n* = 5 mice/group/sex; *n* > 3 NMJs/animal). All data analyzed using 2-way ANOVA and Tukey’s post hoc when appropriate. Error bars show standard deviation. Scale bars: 15 μm.

In addition to atrophy, patients with CKD frequently exhibit impaired muscle strength/function and exercise intolerance (43, 44). In contrast to these clinical observations, the above muscle contractile function experiments showed no impairment in specific force, power, or fatigue in CKD mice. It is important to consider that these functional experiments were performed ex vivo, using direct electric stimulation to depolarize the muscle cell membrane and initiate contraction, thus bypassing the neural transmission through the neuromuscular junction (NMJ). To begin to consider this aspect of the CKD muscle phenotype, comprehensive morphometric analysis of the NMJ was performed by a blinded investigator. Fluorescently conjugated antibodies were used to label the acetylcholine receptor (α-bungarotoxin), presynaptic motor neuron terminals (synaptophysin), and the motor neuron axon (neurofilament 200). Confocal micrographs of control and CKD are shown in Figure 9, A–H. Quantitative analyses of NMJ morphology indicated signs of NMJ stress/remodeling, including decreased number of nerve terminal branches and branch points, axonal swelling (increased nerve terminal area), signs of denervation (unoccupied acetylcholine receptor [AChR] area), and polyinnervation, demonstrating significant group effects of CKD (Figure 9, E–I). These findings suggest that NMJ stress may be a potential factor contributing to muscle fiber atrophy/dysfunction in CKD that warrants future evaluation using in situ assessments of NMJ function.

## Discussion

Hallmarks of the CKD patient include myopathic symptoms, such as muscle weakness, fatigue, atrophy, and exercise intolerance. Much like the damage to the kidneys, these muscle-related symptoms often develop over long periods, and epidemiological and cross-sectional clinical studies have reported strong associations between the severity of myopathy and kidney function (GFR or diagnosed CKD stage) (42–44). To date, much of the CKD literature examining the basis for myopathy has focused on mechanisms of protein degradation (3, 7). Although skeletal muscle mitochondrial alterations have been implicated to play a role in the CKD-associated myopathy (12, 14, 15, 17, 20, 22, 45), previous measures of mitochondria have been limited to enzyme activity, content measures, and traditional oxygen consumption assessments. Thus, a comprehensive understanding of the impact of CKD on skeletal muscle mitochondria is lacking. To fill this void in the literature, this study employed a comprehensive mitochondrial phenotyping platform (33, 34) to uncover the impact of CKD on muscle mitochondrial energetics. OXPHOS conductance, assessed using a potentially novel energetic clamp to mimic physiologically relevant levels of cellular energy demand, was decreased by approximately 30% in CKD mice compared with mice with healthy renal function. To help delineate the basis of the impaired mitochondrial function, a platform of biochemical experiments determined that the decrease in OXPHOS was caused by decreased maximal activity of matrix dehydrogenases (PDH, AKGDH, BCKDH), rather than the enzymes of the ETS or ATP synthase (Figure 5). Consistent with biochemical findings herein, recent studies have also reported that both mice (5/6 nephrectomy) and human CKD patients have decreased PDH activity in skeletal muscle (20, 46), suggesting common mechanisms that converge on mitochondrial dehydrogenases. Additional metabolomics profiling of skeletal muscle, including pathway enrichment analysis, identified disruptions in amino acid metabolism and elevated uremic toxins, particularly in male CKD mice. These metabolic changes were coincident with increased muscle protein degradation and muscle atrophy but normal contractile performance in ex vivo muscle preparations.

Although impaired mitochondrial respiration has been previously reported in both preclinical CKD models and human patients (14, 15, 17, 19, 21), the molecular mechanisms underlying these changes have not been defined. In this study, a comprehensive mitochondrial phenotyping analysis identified matrix dehydrogenase as the principal site of impaired energy transduction. Interestingly, the dehydrogenases most affected by CKD (PDH, BCKDH, AKGDH) all have similar structural characteristics including 3 catalytic components — E1, E2, and E3 — and require coenzymes thiamine pyrophosphate, coenzyme A, and NAD^+^ for the reaction to be catalyzed. The structural similarity of these dehydrogenases implies that a unified biochemical mechanism may exist. Both PDH and BCKDH are regulated by phosphorylation at serine residues on the E1 catalytic site. A recent study reported that pyruvate dehydrogenase kinase-4, a negative regulator of PDH activity, was upregulated in muscle of patients with end-stage renal disease (46). In contrast, BCKDH and AKGDH have not been studied in CKD muscle to date to our knowledge. Based on the findings herein, additional studies are needed to uncover the biochemical mechanisms contributing to impaired dehydrogenase activity in CKD muscle.

This study reports a substantial impairment on mitochondrial OXPHOS function in skeletal muscle of CKD mice. The clinical impact of decreased mitochondrial energy transduction may help explain the observed exercise intolerance and decreased physical function in CKD patients (21, 24, 44, 47–49). With initiation of contractile activity, the increased ATP hydrolysis by contractile proteins (myosin ATPase) causes a rapid decrease in the cellular energy charge (ATP/ADP), which results in a decrease in the available free energy for ATP hydrolysis (ΔG_ATP_), which reduces the available power for the contractile work. From a bioenergetic perspective, mitochondria’s role is to respond to this decrease in extramitochondrial ΔG_ATP_ by increasing OXPHOS to maintain sufficient energy charge to sustain contractile activity. Mitochondrial dysfunction has been linked to muscle atrophy for many years (50). Over the past couple of decades, much of this link has focused on the role of mitochondrion-derived ROS (51). In the current study, mitochondrial ROS and electron leak were not altered in CKD muscle under conditions mimicking resting skeletal muscle, suggesting mitochondrial ROS are not causal in the development of muscle atrophy in CKD. A potential mechanistic link may involve energetic stress (i.e., reduced energy charge), which has been previously shown to activate AMP kinase/FoxO3 pathways and contribute to muscle atrophy (52). Additionally, several studies have reported fiber type-specific susceptibility to atrophy. Type 1 (slow oxidative) muscle fibers with higher mitochondrial content appear to have more severe reductions in size and overall number of fibers, implying another link between mitochondria and atrophy. Nonetheless, future mechanistic work targeting mitochondrial energetic stress is still needed to establish causality in regard to muscle atrophy.

Interestingly, significant correlations between kidney function (GFR) and muscle mitochondrial OXPHOS conductance were observed in all substrate conditions when considering all mice in the current study (Figure 2F). Likewise, approximately 100 metabolite features that were found to be elevated in CKD muscle were inversely correlated (*P* < 0.1 was used because of the limited size of metabolomics analyses) with OXPHOS function and consequently directly associated with GFR (Figure 7). Because a major function of the kidneys is to filter and remove many metabolic waste products, CKD and the consequent uremic accumulation of metabolites could be potential candidates for systemic mediators of mitochondrial alterations. Several uremic metabolites were substantially elevated in skeletal muscle of CKD mice, including l-kynurenine, indole-3-acetate, 3-indoleacetonitrile, aminoadipic acid, dimethylarginine, and trimethylamine-*N*-oxide. Consistent with metabolomics analyses in the current study, a previous study also reported higher levels of trimethylamine-*N*-oxide, allantoin, aminoadipic acid, trimethyllysine, and dimethylarginine in skeletal muscle using the 5/6 nephrectomy model in mice (53). Indole-3-acetate and l-kynurenine were recently shown to impair skeletal muscle mitochondrial ETS and select matrix dehydrogenases (34). Additionally, kynurenine and tryptophan-derived indole metabolites have been shown to decrease mitochondrial function (in vitro) in heart (38, 39), kidney epithelial cells (54), liver (37), and skeletal muscle (55). These observations lend support to a hypothesis of uremic myopathy whereby muscle function and mitochondrial health may be related to the progressive accumulation of uremic metabolites consequent to GFR insufficiency (44, 48, 56).

Previous studies have reported that mice and humans with CKD displayed lower mitochondrial content/density in skeletal muscle (14, 20, 46, 57), which contrast the observations herein. There are several biomarkers used to estimate mitochondrial content in muscle; however, they vary widely in accuracy when compared with electron microscopy (a gold standard) (58). In the present study, 3 of the best biomarkers of mitochondrial content (citrate synthase, complex I, and complex IV activity) were not different between control and CKD mice. The reasons for these contrasting findings are likely multivariate because the choice of muscle, method of CKD induction, and method of assessing mitochondrial content vary across these studies. For instance, Tamaki et al. demonstrated reduced mitochondrial amount and reduced PGC-1α gene expression in quadriceps muscle from CKD mice (20). However, this study utilized a nephrectomy model and performed assays in a muscle that characteristically has more type II glycolytic fibers than red gastrocnemius, and mitochondrial content was assessed using a fluorescent dye (Mito Tracker Green FM). Moreover, Gamboa et al. reported reduced mitochondrial content using electron microscopy analysis of muscle biopsies as well as mitochondrial DNA content (14); however, these data were from patients with stage 5 (severe) CKD. Despite these contrasting results, given the data obtained from numerous biomarkers performed in the present study, we conclude that mitochondrial content does not change in muscle from adenine-fed mice.

Oxidative stress is often suspected to be a contributing factor to the muscle wasting phenotype in CKD (14, 22, 59, 60) given the redox-sensitive nature of atrophy signaling pathways in skeletal muscle (51). Surprisingly, this study did not detect physiologically significant increases in mitochondrial ROS (H_2_O_2_) production in skeletal muscle (Figure 3). With mitochondria placed under state 2 conditions (maximal substrate with no ATP demand), there were statistically significant elevations in mitochondrial electron leak supported by pyruvate/malate and glutamate/malate, as well as trending in FA metabolism (octanoyl-carnitine/malate). However, these conditions are nonphysiological because skeletal myofibers will always have some level of energy demand. When placing the same mitochondria in conditions with resting levels of energy demand, the elevated electron leak in CKD muscle was abolished. These findings are in opposition to those reported in a recent study (61) using a nephrectomy model in young male Wistar rats. This latter study reported approximately 30% increase in mitochondrial H_2_O_2_ production under several substrate conditions; however, all experiments were performed in the absence of an energy demand (state 2). Rats in this prior study also exhibited increased ratio of oxidized glutathione and reduced glutathione in skeletal muscle, which were not found in the muscle metabolomics data in the present study (see Supplemental Table 1). Despite our not observing a physiologically relevant level of mitochondrial ROS, some features present in the metabolomics data suggest nonmitochondrial oxidative stress may be present in skeletal muscle of CKD mice. For example, CKD mice exhibited an approximately 2-fold increase in allantoin, a metabolic product of uric acid oxidation that involves numerous ROS/reactive nitrogen species. Muscle from CKD mice also displayed lower concentrations (approximately 15%–25% decrease) of several histidine-containing dipeptides (carnosine, sarcosine, anserine) that have antioxidant properties (62, 63). Additionally, CKD muscle exhibited an approximately 60% decrease in ergothioneine (a thiol-based dietary antioxidant, ref. 64) levels. Taken together, these results suggest that future studies are necessary to elucidate the role of nonmitochondrial ROS in CKD-associated skeletal myopathy.

Consistent with the literature, adenine-fed mice had reduced muscle mass and myofiber CSA compared with their counterparts with normal kidney function (Figure 8). Ex vivo assessments of solei muscles supported that adenine-fed mice displayed increased rates of protein degradation compared with controls, a phenotype consistent with findings from mice subjected to the 5/6 nephrectomy CKD model (41, 65) (Figure 8E). Although muscle atrophy is commonly reported in both the preclinical and clinical CKD literature, few studies have systematically evaluated the functional impact. To investigate the functional impact of CKD on skeletal muscle, contractile function was assessed in soleus muscles using isometric (peak force) and shortening (peak power) contractions as well as muscle fatigue. Peak absolute force production was decreased in male CKD mice; however, when normalized to muscle weight, the specific force was unaffected in CKD mice. Furthermore, there were no impairments in either peak power or muscle fatigue in CKD mice. Our findings are consistent with a recent study involving a genetic rat model of polycystic kidney disease (Cy/+) where 35-week-old rats had normal plantarflexion function in vivo (although interestingly, dorsiflexion was impaired) (66). Contrasting the lack of contractile deficits at the whole-muscle level, Mitrou et al. (67) reported decreased maximal isometric force in single permeabilized psoas fibers from New Zealand rabbits with surgically induced CKD (12 weeks in duration). It is difficult to draw strong conclusions from these studies because each study involved a different species (mouse, rat, rabbit) and different model of CKD (adenine, Cy/+, surgical) and had different functional experiments (whole muscle ex vivo, in vivo, vs. single fiber). Nonetheless, the results presented herein suggest that CKD-mediated changes in muscle function are driven entirely by atrophy or take longer than 10 weeks to develop in mice or originate from pathological changes proximal to the myofiber (i.e., motor neuron).

Although contractile deficits in skeletal muscle were not present in the current study, numerous human studies have demonstrated impaired muscle function in CKD patients (47, 48, 68). There are several logical explanations for these seemingly contrasting findings. The contractile assessments herein were performed in ex vivo muscle preparations wherein direct electrical stimulation was applied to depolarize the sarcolemma, thus bypassing the neuromuscular transmission involved in voluntary muscle contractions. Indeed, morphological assessment of the NMJ in CKD skeletal muscle revealed signs of destabilization of the NMJ, including less nerve terminal branching, as well as axonal swelling and signs of polyinnervation (Figure 9). Persistent NMJ destabilization is believed to accelerate muscle atrophy and likely impair in vivo muscle contractility (69). Functional evidence supporting NMJ abnormalities can be found in a recent study in CKD patients that reported impairments in muscle strength, balance, and fine motor skills (Moberg’s picking up test) (48). Future work is needed to expand the understanding of the impact of NMJ remodeling on CKD-associated skeletal myopathy.

Although not a primary goal of the current study, the present data unexpectedly uncovered sex-specific differences in the murine CKD myopathy. Confirming a previous report (26), female mice on adenine in the present study had a modest, but statistically insignificant, increase in BUN. Despite this, female CKD mice had reduced GFRs and renal histological changes (e.g., interstitial inflammation) similar to male CKD mice. Further, female mice on adenine displayed lower kidney fibrosis, and there were notable differences in metabolite profiles in skeletal muscle from male and female mice with CKD. For example, indole-3-acetate and 3-indoleacetonitrile were increased in male but not female CKD mice. There were also marked differences in the levels of dimethylarginine and trimethylamine-*N*-oxide in skeletal muscle of male and female CKD mice (males having greater increases). The mechanisms underlying these sex differences are currently unknown. To date, the vast majority of preclinical CKD research has been performed on young male animals. Estrogen has been reported to have renoprotective properties (26, 70) and has been shown to affect skeletal muscle biology and mitochondrial energetics (71, 72). In this regard, female mice in the current study were of young age and were not ovariectomized, a phenotype that is not exactly representative of the typical female CKD patient, who is likely to be postmenopausal.

Although there were no statistical effects of sex on mitochondrial bioenergetics in the current study, female CKD mice notably displayed more modest impairments in OXPHOS compared with their male counterparts (approximately 20% decrease in females vs. approximately 35% decrease in males), as well as differences in metabolite abundance in muscle. Nevertheless, future work should carefully consider sex differences, and more studies are needed to fully discern the impact of sex hormones in CKD-associated myopathy and uremia.

There are several limitations of the current study. First, experiments were carried out in young male and normally cycling female mice, which do not model the CKD patient well. While kidney function begins to naturally decline at approximately 40 years of age, the prevalence of CKD increases markedly above age 60 (73, 74), an age where most female CKD patients are postmenopausal. It is unknown if using aged mice would result in acceleration or worsening of the myopathy condition in CKD. Future studies using female mice should consider performing oophorectomy to better mimic the female CKD patient, which may produce different findings than the current study, particularly related to the susceptibility to muscle atrophy (72). Another important consideration is that the current study was performed on mice that were housed at standard room temperature (23°C), which is below thermoneutrality. Recent studies have documented profound physiological and metabolic differences in mice housed at thermoneutral temperatures compared with housing at ambient temperatures (75, 76), including lower basal energy expenditure (due to need for thermogenesis), and future work investigating metabolic complications resulting from CKD should consider housing/body temperature. As with many metabolomics studies, a number of metabolites featured with statistically significant abundances in CKD were unidentifiable (i.e., not present in the in-house library or reliably found in metabolomics databases). Therefore, the relative biological importance is unknown, and future work is needed to identify these features using NMR. Another limitation for consideration is the fact that the adenine model, similar to surgical CKD models, causes a rapid decline in GFR, which is not present in most CKD patients. Moreover, dietary adenine is not a specific nephrotoxin and thus might affect other tissues/organs. To minimize these potential impacts, mice in this study were placed back on casein diet for 2 weeks before terminal experiments to allow for a washout period. Last, to obtain sufficient mitochondrial yield to complete all the mitochondrial phenotyping experiments on the same animals, many muscles were pooled together, regardless of fiber type, and used for the mitochondrial isolation protocol. Because of this, fiber type-specific impacts of CKD could not be examined, and this tissue requirement limited the available muscles for functional and histological analyses.

In summary, the current study establishes matrix dehydrogenases as the principal site responsible for skeletal muscle mitochondrial dysfunction in the murine CKD model. This dysfunction was characterized by substantial decreases in mitochondrial OXPHOS, which was strongly associated with the level of kidney function. Untargeted metabolomics analysis of skeletal muscle identified numerous potentially novel, unidentified uremic metabolites that accumulated in CKD muscle and strongly associated with the degree of mitochondrial impairment. Additional phenotyping of muscle identified the NMJ as a potential site contributing to the progressive myopathy associated with CKD.

## Methods

Additional detailed methodology can be found in Supplemental Methods.

### Animals.

C57BL/6J mice (stock 000664) were obtained from The Jackson Laboratory at 8 weeks of age (*N* = 61 total mice). All rodents were housed in a temperature- (22°C) and light-controlled (12-hour light/12-hour dark cycle) room and maintained on standard chow before CKD induction with free access to food and water.

### Induction of CKD.

We utilized an established adenine diet model (27, 77–81) to induce CKD in mice. Mice were assigned to a casein-based chow diet for 7 days, followed by induction of renal tubular injury by supplementing the diet with 0.2% adenine for 7 days, and were subsequently maintained on a 0.15% adenine diet for 7 more weeks. CKD mice were then placed back on control casein diet for 2 weeks before euthanasia and terminal experiments. Control mice received casein diet for the duration of the study. Casein-fed mice consumed more food when compared with adenine-fed mice (3.0 ± 0.3 vs. 2.5 ± 0.4 g/d in males; 2.4 ± 0.3 vs. 2.1 ± 0.3 in females, *P* = 0.047). Detailed information about the diet composition can be found in Supplemental Table 1.

### Assessment of kidney function.

GFR was measured using FITC-inulin clearance as previously described (29, 82, 83). GFR was calculated using a 2-phase exponential decay in GraphPad Prism (29, 82, 83). BUN was also assessed in plasma using a commercial kit (Arbor Assays K024) per the manufacturer’s instructions.

### Kidney histology.

Kidney histology was assessed by standard light microscopy with Masson’s trichrome staining as previously described (29).

### Preparation of mitochondrial isolation.

Skeletal muscle mitochondria were isolated as previously described (34, 84).

### Measurement of oxidative capacity and OXPHOS conductance.

High-resolution respirometry was measured using Oroboros Oxygraph-2k (O2K) measuring oxygen flux (*J*O_2_) at 37°C in buffer Z (105 mM K-MES, 30 mM KCl, 1 mM EGTA, 10 mM K_2_HPO_4_, 5 mM MgCl_2_-6H_2_O, 2.5 mg/mL BSA, pH 7.2), supplemented with either 5 mM creatine (conductance assay) or 20 mM creatine (capacity assay). Traditional respirometry was used to assess oxidative capacity and integrity of the mitochondrial isolation using 10 mM pyruvate + 2 mM malate as substrates, followed by a bolus of ADP (16 mM) and cytochrome *c* (10 mM). Samples with a cytochrome *c* response greater than 15% were excluded from the study (3 samples were excluded). In the OXPHOS conductance assay, a creatine kinase (CK) clamp was employed to leverage the enzymatic activity of CK, which couples the interconversion of ATP and ADP to that of phosphocreatine (PCr) and free creatine, to titrate the extramitochondrial ATP/ADP ratio, and thus free energy of ATP hydrolysis (ΔG_ATP_) was calculated from the added phosphocreatine (PCr)/creatine (Cr) ratio as done previously (33, 34). This approach permits assessment of mitochondrial flux across a range of physiological ATP free energy states, which are set experimentally by altering the Cr/PCr ratio. Overall, the ΔG_ATP_ is plotted against the corresponding *J*O_2_, creating a linear force-flow relationship, where the slope represents the conductance throughout the ETS. In this assay, the ΔG_ATP_ is manipulated through titrations of PCr that result in a corresponding reduction in ADP concentrations (Figure 1C). Mitochondria (25 μg) were added to the O2K chamber in 2 mL of buffer Z supplemented with ATP (5 mM), Cr (5 mM), PCr (1 mM), and CK (20 U/mL). Assessments were done at 37°C. Conductance measurements were performed under the following conditions: pyruvate (5 mM) + malate (2.5 mM), glutamate (10 mM) + malate (2.5 mM), succinate (10 mM) + rotenone (0.005 mM), and octanoyl-carnitine (FA, 0.2 mM) + malate (2.5 mM).

### Mitochondrial H_2_O_2_ production and electron leak.

Mitochondrial H_2_O_2_ production was measured fluorometrically via Amplex UltraRed (AUR)/HRP detection system (Thermo Fisher Scientific, excitation/emission 530:590 nm) as described previously (34). H_2_O_2_ production was measured in buffer Z supplemented with Cr (5 mM), CK (20 U/mL) AUR (10 μM), HRP (1 U/mL), superoxide dismutase (20 U/mL), ATP (5 mM), and auranofin (0.1 μM). Using the CK clamp, experiments were run using 2 levels of energy demand: (a) state 2 maximal H_2_O_2_ production with no addition of PCr and (b) resting ΔG_ATP_ (–15.24 kcal/mol) set by adding 30 mM PCr. H_2_O_2_ production was assessed with the following substrate conditions: pyruvate (5 mM) + malate (2.5 mM), glutamate (5 mM) + malate (2.5 mM), succinate (10 mM), and octanoyl-carnitine (0.2 mM) + malate (2.5 mM). All reactions were done at 37°C, 200 μL of volume containing 20 μg of mitochondria in a BioTek Synergy 2 Multimode Microplate Reader. Rates were collected and converted to pmoles H_2_O_2_/min/mg using a standard curve. Auranofin was added to inhibit the endogenous thioredoxin buffering system within the mitochondrial matrix, therefore allowing a more accurate approximation of total H_2_O_2_ produced under respiratory conditions (33). Fluorescence values were converted to pmoles of H_2_O_2_ via a H_2_O_2_ standard curve. Percentage electron leak was calculated by dividing *J*H_2_O_2_ by *J*O_2_ ([*J*H_2_O_2_/*J*O_2_] × 100) in the 3 substrate conditions in the conductance assay: pyruvate (5 mM) + malate (2.5 mM), glutamate (5 mM) + malate (2.5 mM), and octanoyl-carnitine (0.2 mM) + malate (2.5 mM). Succinate H_2_O_2_ electron leak rates were not calculated due to differences in the assay conditions due to adding rotenone in conductance *J*O_2_ assay to prevent reverse electron flow.

### Assessment of matrix dehydrogenase activity.

The activity of several mitochondrial matrix dehydrogenases (*J*NADH) was measured fluorometrically using NAD(P)H autofluorescence (excitation/emission 340:450 nm) in a 96-well plate read kinetically on a BioTek Synergy 2 Multimode Microplate Reader as previously described (34). Full, uncropped blots can be found in the online supplemental material.

### ATP synthase activity.

ATP synthase activity (complex V) was measured as previously described (33, 34).

### Assessment of mitochondrial content.

Mitochondrial content was assessed using a total OXPHOS rodent Western antibody (1:1000, Abcam ab110413) to determine relative levels of OXPHOS complex subunits in mouse mitochondria and via citrate synthase activity as previously described (84).

### Assessment of mitochondrial OXPHOS complex enzyme activity.

Enzyme activity of all ETS complexes were determined spectrophotometrically as previously described (34).

### Muscle protein degradation.

The rate of protein degradation was measured by release of free tyrosine from incubated muscles in the presence of cycloheximide (a protein synthesis inhibitor) as done previously (41, 85). Briefly, immediately following sacrifice, soleus muscle was excised and wet weight was obtained. The muscles were then pinned through the tendons at resting muscle length in 1× Kreb’s buffer (137 mM NaCl, 5 mM KCl, 10 mM NaH_2_PO_4_, 24 mM NaHCO_3,_ pH = 7.4) supplemented with 10 mM glucose and 0.5 mM cycloheximide and gassed with 95% O_2_ and 5% CO_2_ at 37°C for 30 minutes. After the initial 30-minute incubation, the buffer was replaced with fresh Kreb’s buffer and continuously gassed (95% O_2_ and 5% CO_2_) for 3 hours at 37°C. Next, the Kreb’s buffer was collected, snap frozen in liquid nitrogen, and lyophilized overnight (Labconco Free zone 2.5 L –50°C #700201000). The lyophilized samples were resuspended in a mixture of 50 μL of 50 mM phosphate buffer (pH 7.2) along with 0.5 mM D6-4,4-dimethyl-4-silapentane-1-sulfonic acid as an NMR internal standard, 2 mM EDTA and 0.2 % sodium azide (Chenomx, Inc.) in a deuterated environment. All NMR spectra were acquired at the University of Florida, using a 14.1 T Bruker Avance II Console NMR system (Bruker Biospin) consisting of a 1.7 mm CP TXI CryoProbe. Proton (^1^H) NMR spectra were collected with the first slice of a NOESY pulse sequence (tnnoesy) (86) at room temperature. All the parameters used to acquire 1D NOESY spectra were same as reported previously (87–89), except the number of scans (256 scans here). Quantification of tyrosine was performed using Chenomx NMR Suite 8.2 (Chenomx, Inc.) and normalized by muscle wet weight.

### Immunofluorescence microscopy.

Skeletal myofiber CSA and capillary count were assessed by immunofluorescence microscopy using laminin staining (MilliporeSigma L9393, 1:100). Full methodological details for staining and analysis can be found in Supplemental Methods.

### Assessment of muscle contractile function.

Immediately following sacrifice, the left limb was amputated. The soleus was quickly excised under a stereo-zoom microscope and carefully mounted to a Dual-Mode Muscle Lever System (300C-LR; Aurora Scientific). The distal tendon was attached to a secured glass rod using a loop of suture. We mounted the muscle between 2 platinum electrodes submerged in a water-jacketed organ bath containing bicarbonate-buffered solution at room temperature and continuously gassed with 95% O_2_–5% CO_2_. We adjusted the muscle length to attain maximal twitch tension (optimal length, L_O_), increased the temperature of the organ bath to 32°C, and allowed 10 minutes for thermal equilibration. We then measured isometric forces at stimulation frequencies of 1, 30, 40, 50, and 200 Hz delivered by biphasic high-power stimulator (701C, Aurora Scientific) delivered with current of 600 mA, pulse duration 0.25 ms, and train duration 500 ms. Full methodological details can be found in Supplemental Methods.

### NMJ staining and analysis.

TA muscles were harvested and washed in ice-cold PBS, then fixed overnight in 4% paraformaldehyde at 4°C. The fixed muscle portions were separated into small bundles by gentle dissection using forceps and blocked overnight at 4°C in 5% goat serum, 5% BSA, and 2% Triton X-100 in PBS, then incubated with mouse anti-synaptophysin (1:25; ab8049, Abcam) and rabbit anti–neurofilament 200 (1:200; N4142, MilliporeSigma) overnight at 4°C to label presynaptic motor neuron terminals and axons, respectively. NMJ image stacks were obtained with a Leica SP8 confocal microscope with a 63× objective and analyzed using ImageJ (NIH). NMJ morphology was characterized for en face endplates using the following categories: (a) axon diameter, (b) number of nerve terminal branches, (c) number of nerve terminal branch points, (d) nerve terminal area, (e) percentage unoccupied AChR area, (f) synaptophysin compactness, and (g) end plate area. An average of 10 NMJs per animal were analyzed. All analyses were done by a single observer blinded to the identity of the sample. Full methodological details can be found in Supplemental Methods.

### Serum and skeletal muscle metabolomics.

Metabolomics analyses were performed by the Southeast Center for Integrated Metabolomics (SECIM, http://secim.ufl.edu) at the University of Florida. Raw metabolomics data have been deposited to Metabolomics Workbench (https://www.metabolomicsworkbench.org) with the following study IDs: ST001361 (serum) and ST001353 (skeletal muscle). Under ketamine/xylazine anesthesia, blood was collected from a 1 mm tail snip, allowed to clot for 20 minutes at room temperature, and centrifuged at 4000*g* for 10 minutes. Serum was collected and stored at –80°C until analysis. Red gastrocnemius muscle was dissected and snap frozen in liquid nitrogen and stored at –80°C until analysis. Full methodological details can be found in Supplemental Methods.

Data from positive and negative ion modes were separately subjected to statistical analyses. MZmine (freeware) was used to identify features, deisotope, align features, and perform gap filling to fill in any features that may have been missed in the first alignment algorithm. All adducts and complexes were identified and removed from the data set. The primary source of feature identification was performed by mapping against an internal retention time metabolite library established by the SECIM. Additional metabolite searches were performed using the Human Metabolome Database (http://www.hmdb.ca) and the Metabolomics Workbench through a search of the *m/z* ratio with a [M+H] adduct and a tolerance of ±0.002 *m/z*. Statistical analysis of global metabolomics data was performed using MetaboAnalyst (https://www.metaboanalyst.ca). Peak intensity tables were input to MetaboAnalyst with no filtering or normalization to maximize the number of features analyzed. Principal component analysis and partial least squares discriminant analysis were performed across the 4 groups (male control, male CKD, female control, female CKD), and the top 20 VIP scores were presented. Enrichment network analysis and KEGG pathway analysis were also performed using MetaboAnalyst 4.0. The complete list of metabolomics features and statistical analyses can be found in Supplemental Table 2.

### Statistics.

Data are presented as mean ± SD. Normality of data was assessed using the Shapiro-Wilk test. Data that were not normally distributed were analyzed using a Kruskal-Wallis test. Data were first analyzed using 2-way ANOVA with Tukey’s post hoc multiple comparisons when significant interactions were detected. When sex differences or group × sex interactions were detected, data are presented accordingly. Pearson correlations were performed using 2-tailed statistical testing. All statistical analysis was performed in GraphPad Prism (Version 8.0). *P* < 0.05 was considered statistically significant.

### Study approval.

All animal experiments adhered to the *Guide for the Care and Use of Laboratory Animals* from the Institute for Laboratory Animal Research, National Research Council, Washington, DC, National Academies Press, 1996, and any updates. The Institutional Animal Care and Use Committee of the University of Florida approved all procedures.

## Author contributions

TT, STS, and TER were responsible for conception and design of the study. TT, RAK, SKB, RBK, ZRS, RCK, MDC, DDC, RTH, STS, LFF, and TER were responsible for data acquisition, analysis, and/or interpretation. TT and TER drafted the manuscript. TT, RAK, SKB, RBK, ZRS, RCK, MDC, DDC, RTH, STS, LFF, and TER revised, edited, and approved the manuscript. TER and STS obtained funding to directly support this work.

## Supplementary Material

Supplemental data

Supplemental Tables 1-6

## Version 1 12/08/2020

In-Press Preview

## Version 2 01/11/2021

Electronic publication
