# Supplementary material for: Impaired muscle mitochondrial energetics is associated with uremic metabolite accumulation in chronic kidney disease
Source: JCI Insight. 2020 Dec 8;6(1):e139826. doi: 10.1172/jci.insight.139826 (PMC7821598; doi:10.1172/jci.insight.139826)

Impaired muscle mitochondrial energetics is associated with uremic metabolite accumulation in chronic kidney disease

Trace Thome[1], Ravi A. Kumar[1], Sarah K. Burke[4], Ram B. Khattri[1], Zachary R. Salyers[1], Rachel C. Kelley[1], Madeline D. Coleman[1], Demetra D. Christou[1,3], Russell T. Hepple[4], Salvatore T. Scali[2,5], Leonardo F. Ferreira[1,3], Terence E. Ryan[1,3,#].

[1]Department of Applied Physiology and Kinesiology, [2]Division of Vascular Surgery and Endovascular Therapy, [3]Center for Exercise Science, [4]Department of Physical Therapy, University of Florida, Gainesville, FL. [5]Malcolm Randall Veteran Affairs Medical Center, Gainesville, FL.

Running Title: Mechanisms of muscle mitochondrial dysfunction in CKD

#Correspondence should be addressed to Terence E. Ryan, PhD: 1864 Stadium Rd, Gainesville, FL, 32611. Tel: 352-294-1700 (office); email: ryant@ufl.edu

ADDITIONAL SUPPLEMENTAL MATERIAL AND METHODS

Assessment of Kidney Function. Glomerular filtration rate (GFR) was measured using FITC-inulin clearance as previously described(1-3). Briefly, FITC-inulin was dissolved in 0.9% NaCl (5% w/v) and the solution was dialyzed in the dark for 24h in 0.9% NaCl at room temperature with a 1000-kDa dialysis membrane (Spectrum Laboratories), followed by sterile filtering through a 0.22μm filter (ThermoFisher). FITC-inulin (2μl/g body weight) was injected retro-orbitally under isoflurane. Blood was collected in heparin coated capillary tubes via a ~1mm tail snip at 3, 5, 7, 10, 15, 35, 56, and 75 min following the injection. Blood was centrifuged and plasma was diluted (1:20) and loaded into a 96-well plate along with a FITC-inulin standard curve and fluorescence was detected using a BioTek Synergy II plate reader. GFR was calculated using a two-phase exponential decay in GraphPad Prism(1-3). Blood urea nitrogen (BUN) was also assessed in plasma using a commercial kit (Arbor Assays K024) according to the manufacturer instructions.

Kidney Histology. Kidney histology was assessed by standard light microscopy. Briefly, the kidneys were carefully dissected, and weights were obtained. The left kidney was then placed in optimum mounting medium (OCT) compound and frozen in liquid nitrogen-cooled isopentane for cryosectioning. 5-μm-thick longitudinal sections were cut using a cryotome (Leica CM3050) at -20°C and collected on slides for staining. Masson's Trichrome staining using Weigert's iron hematoxylin was performed and images were collected at 20x magnification using automated image capture/tiling in order to image the entire kidney section using an Evos FL2 Auto microscope (ThermoScientific). All image analysis was performed by a blinded investigator using Image J. Kidney fibrotic area was quantified using ten 20x images per kidney at similar anatomical locations for each animal. Locations included the cortex, medulla, and renal pyramid regions of the kidneys. Images were equally thresholded and quantified as a percent of fibrotic area.

Preparation of Mitochondrial Isolation. Skeletal muscle mitochondria were isolated as previously described(4, 5). Briefly, skeletal muscle was dissected from the tibialis anterior (TA), gastrocnemius, quadriceps, hamstrings, triceps, pectorals, gluteus maximus, and erector spinae muscles and placed in mitochondrial isolation medium (300mM sucrose, 10mM HEPES, and 1mM EGTA, pH=7.1). Dissected muscle was then trimmed and cleaned to remove connective tissue and fat. The muscle was minced on ice, placed in ice-cold mitochondrial isolation medium supplemented with bovine serum albumin (BSA, 1mg/ml), homogenized on ice using a glass-Teflon homogenizer (Wheaton), and subsequently centrifuged at 800xG to pellet non-mitochondrial myofibrillar proteins, nuclei, and other cellular components. The resulting supernatant was centrifuged at 10,000xG to pellet mitochondria. All steps were performed at 4°C. The mitochondrial pellet was gently washed to remove any damaged mitochondria and then re-suspended in mitochondrial isolation medium (without BSA) and protein concentration was determined using bicinchoninic acid protein assay (ThermoScientific #SL256970).

Assessment of Matrix Dehydrogenase Activity. The activity of several mitochondrial matrix dehydrogenases (*J*NADH) were measured fluorometrically using NAD(P)H auto fluorescence (Ex/Em = 340/450) in a 96-well plate read kinetically on a BioTek Synergy 2 Multimode Microplate Reader. For all assays, buffer Z was supplemented with alamethicin (0.03mg/mL), rotenone (0.005mM), NAD^+ or $NADP^+$ (2mM). For pyruvate dehydrogenase, alpha ketoglutarate dehydrogenase, and branched-chain ketoacid dehydrogenase, the assay buffer was supplemented with cofactors Coenzyme A (0.1mM), and thiamine pyrophosphate (0.3mM). For each dehydrogenase, assay buffer was loaded and warmed to 37°C followed by the addition of mitochondria. Mitochondria were permeabilized by alamethicin for five minutes. Dehydrogenase activity was then initiated by addition of the following substrates run in parallel: pyruvate (5mM), malate (5mM), glutamate (10mM), alpha ketoglutarate (10mM), branched-keto-acid-methylvalerate (5mM), or isocitrate (5mM). Rates of NADH production was calculated as the slope of linear portions of the NADH curves. Fluorescence values were converted to pmoles of NADH/NADPH via an NADH/NADPH standard curve.

ATP Synthase Activity. ATP synthase activity (Complex V) was measured as previously described(5, 6). Briefly, mitochondria were incubated with Cell lytic M (Sigma C2978) to lyse mitochondria. Buffer E (HEPES (20mM), KCl (100mM), KH_2PO_4 (2.5mM), $MgCl_2$ $6H_2O$ (2.5mM), and 1% glycerol) was supplemented with lactate dehydrogenase (10mM), pyruvate kinase (10mM), rotenone (0.005mM), phosphoenol-pyruvate (PEP, 5mM), and NADH (0.2mM). NADH levels were determined via auto fluorescence (Ex/Em: 340/450). For this assay, the ATP synthase functions to hydrolyze ATP because lysis of the mitochondria will dissipate their ability to establish a membrane potential (which normally drives ATP synthesis). Using a pyruvate kinase/lactate dehydrogenase coupled assay, ATP hydrolysis by the ATP synthase is coupled to NADH consumption in a 1:1 stoichiometry. Thus, the rate of decrease in NADH auto fluorescence can

be used as a measure maximal ATP synthase activity. Fluorescence values were converted to pmoles of NADH via an NADH standard curve.

Assessment of Mitochondrial Content. Mitochondrial content was assessed using a total OXPHOS rodent western antibody to determine relative levels of OXPHOS complex subunits in mouse mitochondria and via citrate synthase activity. Briefly, snap frozen mouse gastrocnemius was homogenized in cell lytic (Sigma #C2978) in glass Teflon homogenizers and centrifuged at 4,000g for 10 minutes at 4°C. The supernatant was then collected and protein was quantified using a bicinchoninic acid protein assay (ThermoScientific #SL256970). 2x Laemmli buffer (BioRad #161-0737) and β-mercaptoethanol (ACROS #60-24-2) were added and the samples were heated at 52°C for 10 minutes. 10µl of pre stained protein ladder was added to the first well of gel (BioRad #928-60000) and 40µg of each sample was loaded. Gel electrophoresis was run at 80V for 10 minutes, then switched to 100V for 1.5 hours. The gel was imaged for total protein on a Bio Rad imager (GelDoc EZ Imager) and the gel was transferred to a PVDF membrane using a BioRad Trans Blot Turbo system. The PVDF membrane was then imaged for total protein and blocked in blocking solution (Licor #927-60001) for 1-hour at room temperature. Next, the membrane was incubated overnight at 4°C with Total OXPHOS rodent WB antibody cocktail from Abcam (ab110413) at 1:1,000 in blocking solution (Licor #927-60001). After overnight incubation, the membrane was washed 3x10 minutes in TBS+0.01% tween. The membrane was then incubated for 1-hour at room temperature with secondary antibody (Goat anti-mouse IRDye 800CW Licor #926-60001) in blocking solution at 1:15,000. The membrane was washed 3x10 minutes with TBS+0.01% tween and imaged and on a Licor Odyssey CLx. The collected images were analyzed using Image Studio Lite version 5.2 to measure western blot band densities between groups. Citrate synthase activity was assessed using snap frozen gastrocnemius muscle homogenized in Cell Lytic M (Millipore-Sigma Cat #C2978) and spun down at 4,000g for 15 minutes at 4°C. The supernatant was collected and used to assess citrate synthase activity using

a citrate synthase assay kit provided by Millipore Sigma as per manufacturer instructions (Cat# CS0720).

Assessment of Mitochondrial OXPHOS Complexes Activity. Enzyme activity of all electron transport system (ETS) complexes were determined spectrophotometrically as previously described(5). Isolated mitochondria were diluted to 0.5 mg/ml in Cell Lytic M (Millipore Sigma) or hypotonic buffer (25 mM K_2HPO_4, 5.3 mM $MgCl_2$, pH 7.2) and subjected to three freeze-thaw cycles prior to use. Complex I activity was determined in 50 mM potassium phosphate, 3 mg/ml BSA, 240 μM KCN, 4 μM Antimycin A, 50 μM decyl-ubiquinone, and 80 μM 2,6-dichlorophenolindophenol (DCPIP) using 10μg of mitochondria per well in a 96 well plate. NADH oxidation (0.8 mM) was measured through reduction of DCPIP at 600 nm. All complex I activity measurements were corrected for rotenone-insensitive NADH oxidation following the addition of 25 μM rotenone and rates were converted to moles using an extinction coefficient of $19100M^{-1}cm^{-1}$. Complex II (succinate dehydrogenase, SDH) activity was assessed in the following assay buffer: 10 mM KH_2PO_4, 2 mM EDTA, 1 mg/ml BSA at pH = 7.8. Briefly, the assay buffer was supplemented with 0.004 mM rotenone, 0.2mM ATP, 10 mM succinate, and 0.08 mM 2,6-dichlorophenolindophenol (DCPIP). Mitochondria were pre-incubated with 0.2 mM succinate for 30 minutes at room temperature and then 5 μg/well was added to a 96-well plate contained 200μl of assay buffer. The assay was initiated by the addition of oxidized decylubiquinone (0.08mM) and reduction of DCPIP was followed at 600 nm. Rates were converted to moles using an extinction coefficient of $19100M^{-1}cm^{-1}$. As a negative control, malonate was included to inhibit SDH. Complex III activity was measured in 10 mM KH_2PO_4, 2 mM EDTA, 1 mg/ml BSA at pH = 7.8 supplemented with 0.2 mM ATP, 0.24 mM KCN, and 0.11 mM oxidized cytochrome c. Complex III activity was determined kinetically by measuring the reduction of cytochrome c following the addition of reduced decyl-ubiquinone (0.15 mM) at 550 nm using 20μg of mitochondria per well in a 96 well plate. Rates were converted to moles using an extinction

coefficient of $18500 M^{-1}cm^{-1}$. Specificity of the assay was confirmed by the absence of cytochrome c reduction in the presence of myxothiazol (10 μM). Complex IV activity was measured in 10 mM KH_2PO_4, 250 mM sucrose, 1 mg/ml BSA at pH = 6.5 supplemented with 2.5 mM maltoside and 5 μM antimycin A following the addition of 110 μM reduced cytochrome c by following the decrease in absorbance at 550 nm using 3μg of mitochondria per well in a 96 well plate. Potassium cyanide (625 μM) was used to confirm specificity of Complex IV activity.

Immunofluorescence Microscopy. Skeletal myofiber cross-sectional area (CSA) and capillary count were assessed by IF microscopy. 10-μm-thick transverse sections were cut from soleus muscle frozen in liquid nitrogen cooled isopentane in OCT using a Leica 3050S cryotome. Muscle sections were fixed with 4% paraformaldehyde (in PBS) for five minutes at room temperature followed by ten minutes of permeabilization with 0.25% triton X-100 in PBS. After permeabilization, sections were washed with PBS 3 times for 5-minutes. Sections were then blocked for 1hr at room temperature with PBS supplemented with 5% goat serum and 1% BSA and incubated overnight at 4°C with a primary antibody for laminin (Sigma L9393, 1:100) to label the myofibers membrane. Following four washes with PBS, muscle sections were incubated with Alexa-Fluor secondary antibodies (ThermoScientific, 1:250) as well as Dylight594 conjugated Griffonia simplicifolia I isolectin B4 (Vector Laboratories, DL-1207) to label endothelial cells (i.e. capillaries). Coverslips were mounted with Vectashield hardmount containing DAPI (Vector Laboratories, H-1500). Images were obtained at 20x magnification using an Evos FL2 Auto microscope (ThermoScientific). All image analysis of myofibers CSA and capillary density was performed using MuscleJ(7), an automated analysis software developed in Fiji.

Assessment of Muscle Contractile Function. Immediately following sacrifice, the left limb was amputated and transferred to a dish containing ice cold bicarbonate-buffered solution (137 mM NaCl, 5 mM KCl, 1 mM $MgSO_4$, 1 mM NaH_2PO_4, 24 mM $NaHCO_3$, and 2 mM $CaCl_2$) equilibrated

with 95% O2 - 5% CO_2 to maintain pH ~7.4. The soleus was quickly excised under a stereo-zoom microscope. Silk sutures (4-0) were used to tie the proximal tendon to a Dual-Mode Muscle Lever System (300C-LR; Aurora Scientific, Aurora, ON, Canada). The distal tendon was attached to a secured glass rod using a loop of suture. We mounted the muscle between two platinum electrodes submerged in a water-jacketed organ bath containing bicarbonate-buffered solution at room temperature and continuously gassed with 95% O_2 - 5% CO_2. We adjusted the muscle length to attain maximal twitch tension (optimal length, L_O), increased the temperature of the organ bath to 32 °C, and allowed 10 min for thermal equilibration. We then measured isometric forces at stimulation frequencies of 1, 30, 40, 50, and 200 Hz delivered by biphasic high-power stimulator (701C, Aurora Scientific) delivered with current of 600 mA, pulse duration 0.25 ms, train duration 500 ms. Each stimulation train was interspersed by 1 min intervals. Following isometric contractions, we proceeded with a single isotonic release contraction to estimate peak power. Each muscle was maximally stimulated at 200 Hz (450 ms) and allowed to shorten against ~30% of maximal isometric force (50 ms). This load approximately elicits peak power based on the force-power relationship in limb muscles(8, 9). Shortening velocity during the isotonic release was determined at least 10 ms after the initial change in length using high-throughput analysis software (DMA, Aurora Scientific)(10). Power (W/kg) was calculated as the product of shortening velocity (m/s) and load during shortening (N/kg). Muscles were then subjected to a fatigue protocol that consisted of repetitive 40 Hz stimulations interspersed by 1 second for 5 minutes. Muscle stimulation and data collection were controlled through automated software (DMC, Aurora Scientific). Specific force was calculated by normalizing force by muscle weight (grams).

Neuromuscular Junction Staining and Analysis. TA muscles were harvested and washed in ice-cold PBS, then fixed overnight in 4% PFA at 4°C. The fixed muscle portions were separated into small bundles by gentle dissection using forceps and blocked overnight at 4°C in 5% goat

serum, 5% BSA, 2% Triton X-100 in PBS, and then incubated with mouse anti-synaptophysin (1:25 dilution; ab8049, Abcam, Cambridge, MA, USA) and rabbit anti-neurofilament 200 (1:200 dilution; N4142, Sigma, MO, USA) overnight at 4°C to label presynaptic motor neuron terminals and axons, respectively. Muscle bundles were washed in 5% goat serum, 5% BSA in 1xPBS and incubated overnight with AlexaFluor 594-conjugated goat anti-rabbit secondary antibody, AlexaFluor 647-conjugated goat anti-mouse IgG1 secondary antibody, and AlexaFluor 488-conjugated α-bungarotoxin (to identify post-junctional acetylcholine receptor (AChR) clusters; dilution 1:500, B-13422, Life Technologies). Muscle bundles were subsequently washed and then mounted on slides in Prolong Gold Antifade Mountant (ThermoFisher). Neuromuscular junction image stacks were obtained with a Leica SP8 confocal microscope with a 63x objective, and analyzed using ImageJ. Neuromuscular junction morphology was characterized for en face endplates using the following categories: (1) axon diameter; (2) number of nerve terminal branches; (3) number of nerve terminal branch points; (4) nerve terminal area; (5) percent unoccupied AChR area; (6) synaptophysin compactness, and (7) endplate area. An average of 10 NMJs per animal were analyzed. All analyses were done by a single observer blinded to the identity of the sample.

Serum and Skeletal Muscle Metabolomics. Metabolomics analyses were performed by the Southeast Center for Integrated Metabolomics (http://secim.ufl.edu) at the University of Florida. Raw metabolomics data have been deposited to Metabolomics Workbench (https://www.metabolomicsworkbench.org) with the following Study ID's: ST001361 (serum) and ST001353 (skeletal muscle). Under ketamine/xylazine anesthesia, blood was collected from a 1mm tail snip, allowed to clot for 20 minutes at room temperature and centrifuged at 4000xG for 10 min. Serum was collected from stored at -80C until analysis. Gastrocnemius muscle was dissected and snap frozen in liquid nitrogen and stored at -80C until analysis. Full methodological details for metabolomics analyses can be found in the Supplemental Methods and Data.

Serum was processed using a targeted metabolomics platform for quantifying tryptophan metabolites as a number of these metabolites are well establish uremic toxins. Twenty-five microliters of each mouse serum was spiked with 5 µL internal standards (IS) solution consisted of tryptophan $^{13}C_{11}$, serotonin D4, kynurenine D4, kynurenic Acid D5, xanthurenic acid D4, anthranilic acid $^{13}C_6$, indoxyl sulfate $^{13}C_6$, p-cresol sulfate D7 and 3-indole-acetate D7. Metabolite extraction was done by protein precipitation using 200 µl of 8:1:1 Acetonitrile: Methanol: Acetone with 0.1% formic acid. Further protein precipitation was allowed by incubating the samples at 4°C for 20 min. Samples were placed in an ultrasonic bath for 10 min and then centrifuged at 20 000 xg for 5min at 4°C to pellet the protein. 190 µl supernatant was transferred from each sample into clean tube and dried under a gentle stream of nitrogen at 30°C. The dried extracts were re-suspended with 25 µL water with 0.1% formic acid. Resuspension was allowed at 4°C for 10 -15 min then samples were centrifuged at 20000 xg for 5 min at 4°C. Supernatants were transferred into clean LC-vials for targeted LC-MS quantitation on a Thermo Q-Exactive Oribtrap mass spectrometer with Dionex UHPLC and autosampler. All samples were analyzed in positive and negative heated electrospray ionization for all with a mass resolution of 35,000 at m/z 200 as separate injections. Tryptophan, serotonin, kynurenine, kynurenic acid, xanthurenic acid and anthranilic acid were quantified in the positive ionization while indoxyl sulfate, p-cresol sulfate and 3-indole-acetate were analyzed in negative ionization. Separation was achieved on an ACE 18-PFP 100 x 2.1 mm, 2 µm column using a gradient with mobile phase A as 0.1% formic acid in water and mobile phase B as acetonitrile. The flow rate was 350 µL/min with a column temperature of 25°C and injection volume of 2 µL. Run time was 20.5 min. A 9-point calibration curve and QC samples were prepared for targeted quantitation of tryptophan, serotonin, kynurenine, kynurenic acid, xanthurenic acid, anthranilic acid, indoxyl sulfate, p-cresol sulfate and 3-indole-acetate. 20 µL of each calibrator and QCs were supplemented with 5 µl indoxyl sulfate. Peak areas of each analyte and corresponding internal standard in the calibrator, QCs and

samples were integrated using Xcalibur 4.0. A calibration curve was generated by plotting nominal concentration of the analyte in the calibrators versus peak area ratio of analyte and IS. QCs and samples were quantitated against the calibration curve.

Skeletal muscle was processed for Global metabolomics profiling. Briefly, red gastrocnemius muscles were thawed on ice, weighed, and homogenized with a Teflon-tipped conical pestle with a metal rod (Micro-Tube Sample Pestle with Conical Teflon Tip, fits 1.5ml Tubes, autoclavable at 121°F; Research Products International Corp; 199221; Fisher Scientific). The pestle was rinsed with 2-Propanol, water, and methanol and patted dry with a KimWipe in between samples. The samples were centrifuged to pellet the tissue debris and protein concentrations were quantified on the QuBit. The samples were normalized to 500µg/mL of protein with 5mM Ammonium Acetate in water prior to extraction for a total volume of 100µL. 25µL of sample was aliquoted into a clean tube and extracted with 5µL of Global Metabolomics IS and 200µL of 8:1:1 Acetonitrile:Methanol:Acetone to precipitate proteins. The samples were incubated at 4°C for 30 min and centrifuged at 20,000xG at 4°C for 10min. 200µL of supernatant was transferred to a clean Eppendorf tube and dried under nitrogen gas at 30°C and then reconstituted at 25µL in Global Metabolomics Inj. Std. Mix. Next, the samples were processed on a Thermo Q-Exactive Oribtrap mass spectrometer with Dionex UHPLC and autosampler. All samples were analyzed in positive and negative heated electrospray ionization with a mass resolution of 35,000 at m/z 200 as separate injections. Separation was achieved on an ACE 18-pfp 100 x 2.1 mm, 2 µm column with mobile phase A as 0.1% formic acid in water and mobile phase B as acetonitrile. This is a polar embedded stationary phase that provides comprehensive coverage, but does have some limitation is the coverage of very polar species. The flow rate was 350 µL/min with a column temperature of 25°C. 4 µL was injected for negative ions and 2 µL for positive ions. Data from positive and negative ion modes were separately subjected to statistical analyses. MZmine (freeware) was used to identify features, deisotope, align features and perform gap filling to fill in

any features that may have been missed in the first alignment algorithm. All adducts and complexes were identified and removed from the data set. The primary source of feature identification was performed by mapping against an internal retention time metabolite library established by the SECIM. Additional metabolite searches were performed using HMDB (http://www.hmdb.ca) and the Metabolomics Workbench (https://www.metabolomicsworkbench.org) through a search of the *m/z* ratio with a [M+H] adduct and a tolerance of ±0.002 *m/z*. Statistical analysis of global metabolomics data was performed using MetaboAnalyst (https://www.metaboanalyst.ca). Peak intensity tables were input to MetaboAnalyst with no filtering or normalization to maximize the number of features analyzed. Principal Component Analysis and Partial Least Squares Discriminant Analysis were performed across the four groups (male control, male CKD, female control, female CKD) and the top 20 VIP scores were presented. Enrichment network analysis and KEGG pathway analysis was also performed using MetaboAnalyst 4.0. The complete least of metabolomics features and statistical analyses can be found in Supplemental Dataset 1.

REFERENCES

1. Qi Z, Whitt I, Mehta A, Jin J, Zhao M, Harris RC, Fogo AB, and Breyer MD. Serial determination of glomerular filtration rate in conscious mice using FITC-inulin clearance. *American journal of physiology Renal physiology.* 2004;286(3):F590-6.
2. Rieg T. A High-throughput method for measurement of glomerular filtration rate in conscious mice. *J Vis Exp.* 201375):e50330.
3. Berru FN, Gray SE, Thome T, Kumar RA, Salyers ZR, Coleman M, Dennis L, O'Malley K, Ferreira LF, Berceli SA, et al. Chronic kidney disease exacerbates ischemic limb myopathy in mice via altered mitochondrial energetics. *Sci Rep.* 2019;9(1):15547.
4. Ryan TE, Schmidt CA, Green TD, Spangenburg EE, Neufer PD, and McClung JM. Targeted expression of Catalase to Mitochondria Protects Against Ischemic Myopathy in High Fat Fed Mice. *Diabetes.* 2016.
5. Thome T, Salyers ZR, Kumar RA, Hahn D, Berru FN, Ferreira LF, Scali ST, and Ryan TE. Uremic metabolites impair skeletal muscle mitochondrial energetics through disruption of the electron transport system and matrix dehydrogenase activity. *Am J Physiol Cell Physiol.* 2019.
6. Fisher-Wellman KH, Davidson MT, Narowski TM, Lin CT, Koves TR, and Muoio DM. Mitochondrial Diagnostics: A Multiplexed Assay Platform for Comprehensive Assessment of Mitochondrial Energy Fluxes. *Cell reports.* 2018;24(13):3593-606 e10.

7. Mayeuf-Louchart A, Hardy D, Thorel Q, Roux P, Gueniot L, Briand D, Mazeraud A, Bougle A, Shorte SL, Staels B, et al. MuscleJ: a high-content analysis method to study skeletal muscle with a new Fiji tool. *Skelet Muscle.* 2018;8(1):25.
8. Brooks SV, Faulkner JA, and Mccubbrey DA. Power Outputs of Slow and Fast Skeletal-Muscles of Mice. *Journal of Applied Physiology.* 1990;68(3):1282-5.
9. Hill AV. The heat of shortening and the dynamic constants of muscle. *Proc R Soc Ser B-Bio.* 1938;126(843):136-95.
10. Kelley RC, McDonagh B, and Ferreira LF. Advanced aging causes diaphragm functional abnormalities, global proteome remodeling, and loss of mitochondrial cysteine redox flexibility in mice. *Exp Gerontol.* 2018;103(69-79.

Supplemental Table 1: Diet Composition Details

	**Casein Diet**	**0.2% Adenine**	**0.15% Adenine**
**Study Purpose**	Control diet	CKD induction (7d)	CKD maintenance
**Envigo Cat. #**	TD.130898	TD.130900	TD.130899
**Ingredient**	**Amount (g/kg)**		
Casein	200.000	200.000	200.000
DL-Methionine	3.000	3.000	3.000
Corn Starch	392.408	390.408	390.908
Maltodextrin	140.000	140.000	140.000
Sucrose	92.300	92.300	92.300
Corn Oil	50.000	50.000	50.000
Cellulose	50.000	50.000	50.000
Mineral Mix, Ca-P Deficient (79055)	13.370	13.370	13.370
Sodium Phosphate, dibasic	18.500	18.500	18.500
Calcium Phosphate, dibasic, dihydrate	20.000	20.000	20.000
Calcium Carbonate	3.400	3.400	3.400
Vitamin Mix, AIN-76A (40077)	15.000	15.000	15.000
Choline Bitartrate	2.000	2.000	2.000
Thiamin (81%)	0.010	0.010	0.010
Vitamin K_1, phylloquinone	0.002	0.002	0.002
TBHQ, antioxidant	0.010	0.010	0.010
Adenine		2.000	1.5

Supplemental Figure 1 – Uncropped Blots

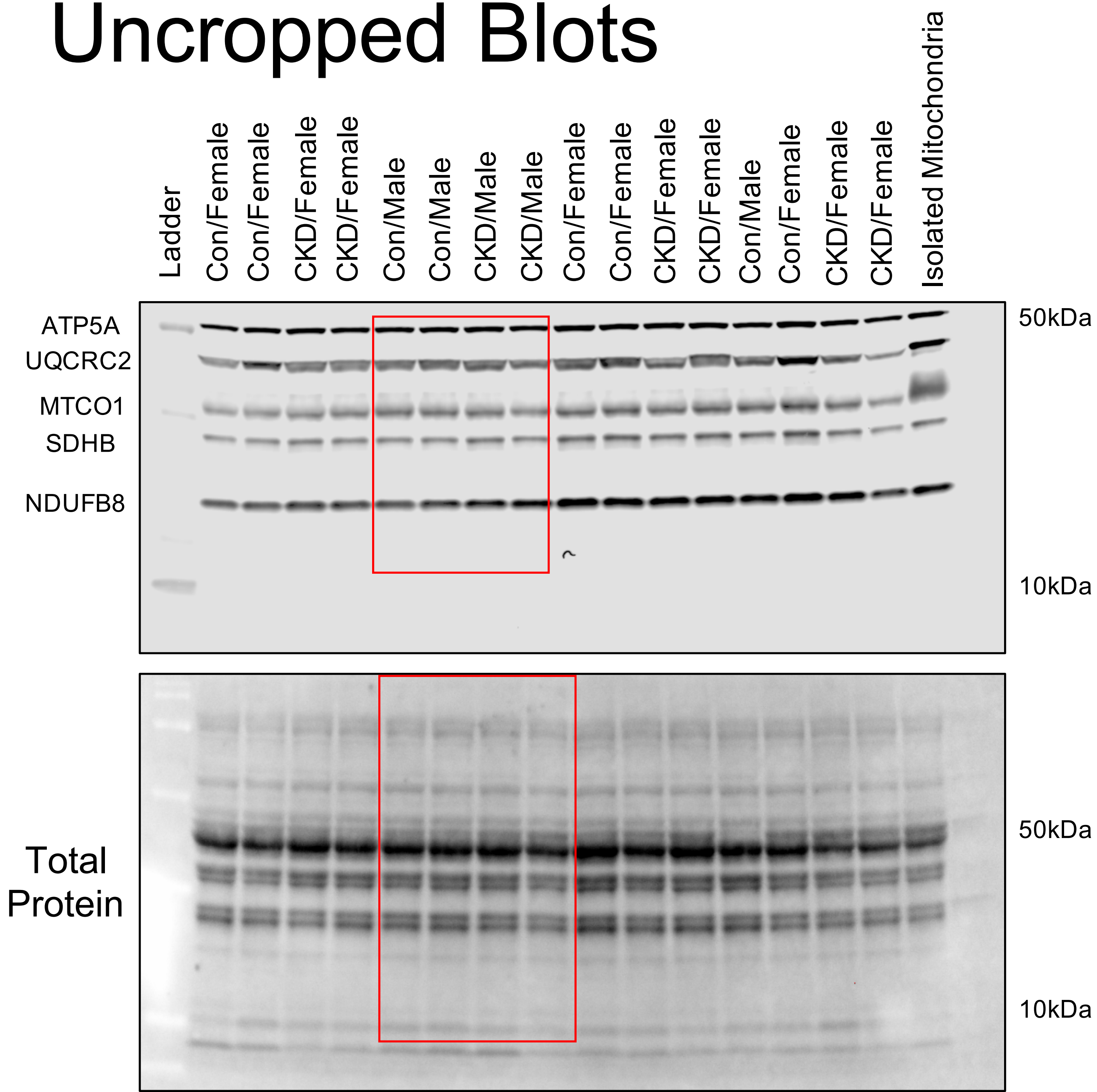

Supplement: Supplemental data [file jciinsight-6-139826-s027.pdf]
